# Companion Diagnostics (CDx) Based on Molecular Biology Techniques

**DOI:** 10.3390/life14111358

**Published:** 2024-10-23

**Authors:** Su Lim Kang, Ji Yean Kwon, Sung Min Kim

**Affiliations:** 1 Department of Medical Device and Healthcare, Dongguk University-Seoul 26, Pil-dong 3-ga, Jung-gu, Seoul 04620, Republic of Korea; slkang2047@gmail.com (S.L.K.); jykwon@dongguk.edu (J.Y.K.); 2 Department of Regulatory Science for Bio-Health Medical Device, Dongguk University-Seoul 26, Pil-dong 3-ga, Jung-gu, Seoul 04620, Republic of Korea

**Keywords:** companion diagnostics, molecular diagnosis, safety, effectiveness, regulatory

## Abstract

Molecular profiling based on genomic mutations provides clinically important diagnostic and prognostic information. Companion diagnostic (CDx) testing, which is based on targeted drug therapy, is being applied to a variety of molecular diagnostic techniques (e.g., fluorescent in situ hybridization—FISH; polymerase chain reaction—PCR; and next-generation sequencing—NGS) to diagnose complex etiologies using a minimal number of specimens, replacing immunohistochemical analysis, which may show bias at certain stages. The safety and effectiveness of CDx testing using molecular diagnostic technology in precision medicine is an important factor in determining the treatment outcome and prognosis of patients. Meeting minimum safety and effectiveness performance standards is essential for CDx testing, and a thorough understanding of regulatory considerations is necessary to plan and design the optimal product. In this review, we focus on the diagnostic field of precision medicine and discuss the safety and effectiveness that each molecular diagnostic technology must meet according to CDx testing diversity.

## 1. Introduction

**Figure 1:**
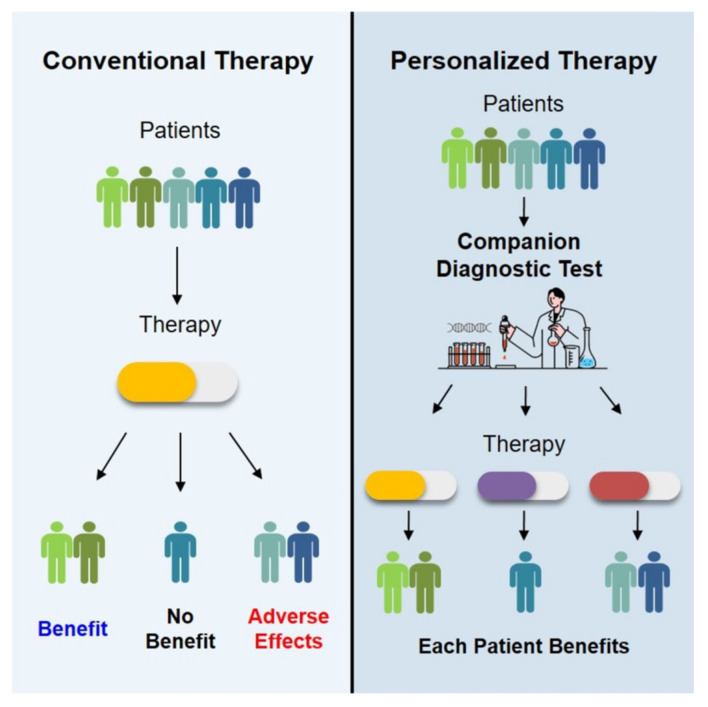
Principles of CDx testing.

Molecular profiling based on the indication of genomic mutations provides clinically important diagnostic and prognostic information. In particular, oncological treatment is being approached by targeting specific treatments to identify the genomic complexity of cancer [1]. Before applying a targeted drug, a companion diagnostic (CDx) test is required to identify the genomic mutation that serves as the target (Figure 1) [2].

A CDx is defined as an in vitro diagnostic device that provides essential information for the safe and effective use of specific therapeutics according to the United States Food and Drug Administration (US FDA) guidelines published in 2014 [3]. The effectiveness of a drug treatment is determined through a CDx compatibility test using molecular diagnostic techniques, and a targeted anticancer drug or personalized anticancer drug is prescribed. Currently, the field in which the joint development of a CDx and drugs is most active is the field of new anticancer drugs, and accordingly, it is widely applied to oncology-related indications [4]. In the early stages of CDx development, immunohistochemical analysis (IHC) technology was applied [5]. IHC analysis can detect specific antigens in histological samples by combining specific antibodies and fluorescent reagents to identify the location of protein expression or lack of expression in lesions. [6]. Although IHC analysis is frequently used to diagnose tumor subtypes in cancer tissues and is an important adjunct technique, it has certain limitations in accuracy and reproducibility [7]. In particular, cases of response bias and ambiguity at certain stages, such as tissue fixation and pretreatment, false-positive antibody reactions, antigen retrieval, and interpretation bias in result analysis, may occur [8,9]. Molecular diagnostic technology has been applied to overcome these limitations and quickly identify the etiology of complex diseases using a minimal number of samples [10], and many products are being developed.

CDx molecular diagnostic technology can be broadly classified into fluorescence in situ hybridization (FISH), polymerase chain reaction (PCR), and next-generation sequencing (NGS). FISH is a technology that hybridizes the nuclear DNA of metaphase chromosomes attached to interphase cells or tumor fragments with a fluorescently labeled nucleic acid probe [11]. Probes are either indirectly labeled with haptens or directly labeled by incorporating fluorophores. The labeled probe and target DNA are denatured and mixed into a single strand, which binds to the complementary DNA sequence. If the probe is indirectly labeled, the visualization of non-fluorescent haptens requires an additional step to incorporate enzymes (fluorochromes that emit a colored signal at the hybridization site) or immunological detection systems, which use light microscopy and ultraviolet light based on the binding of antibodies to specific antigens. Finally, an immunohistochemical reaction that yields a colored reaction is produced [12]. PCR is a technique used to amplify specific DNA fragments in a simple enzymatic reaction that is similar to a laboratory form of DNA replication, a physiological process that uses all living cells to replicate genetic material prior to cell division. PCR involves a DNA template (DNA containing the target sequence), DNA polymerase (an enzyme that synthesizes a new DNA strand complementary to the target sequence), and a primer (a short DNA strand that locates the specific target DNA location and binds through complementary nucleotide base pairing when cooled). The PCR process involves repeated heating and cooling cycles of the nucleotide-containing mixture. The PCR cycle consists of three stages (denaturation, annealing, and extension), and the amount of DNA amplification product increases in each cycle by 2n [13]. The PCR technique has been developed in phases: PCR (first generation), real-time PCR (rtPCR; second generation), and digital PCR (ddPCR; third generation). Second-generation rtPCR, which compensates for the difficulties of quantitative analysis, is widely used, but droplet digital PCR (ddPCR), which amplifies 20,000 droplets for high sensitivity, is also widely used [14,15]. NGS uses fine, spatially separated DNA templates and is a method that decodes vast amounts of genomic information by decomposing a single genome into several pieces, reading each piece simultaneously, and then combining them using computational technology [16]. The NGS analysis process consists of three steps: sample preparation, clonal amplification, and sequencing. NGS technologies comprise four categories: (1) pyrosequencing (detection of inorganic pyrophosphate light energy released during polymerization), (2) microelectrophoresis (electrophoretic separation of DNA fragments), (3) hybridization sequencing (determining the DNA sequence by hybridizing with target DNA and then binding to a complementary probe), and (4) the real-time observation of single molecules (measuring fluctuations in the electrical signal that occurs while DNA passes through a nanopore) [17,18].

Guidance documents and legislation on CDx technologies exist around the world and are evolving. Until recently, major CDx-related guidelines or law revisions have been centered around six major regulatory authorities, and efforts have been made to prepare CDx-specific guidelines in countries such as Canada, the United Kingdom, and Singapore (Supplementary Material Table S1). CDx technology approved by each regulatory agency is shown in Supplementary Material Table S2.

The safety and effectiveness of CDx testing using molecular diagnostic technology is an important factor in determining the treatment outcome and prognosis of patients. Accurately identifying a specific genetic sequence is necessary for managing patients; therefore, the sensitivity and specificity of diagnostic devices and tools are very important [19]. Therefore, it is essential for CDx technologies to meet minimum safety and effectiveness performance standards, and a thorough understanding of regulatory considerations is necessary to plan and design the optimal product [20]. This review discusses the safety and effectiveness of CDx technologies using molecular diagnostic techniques (FISH, PCR, and NGS), focusing on the diagnostic field of precision medicine. In addition, CDx products approved by regulatory agencies are analyzed to emphasize discussions on the regulatory environment in major countries.

## 2. Methods

### 2.1. Research Scope

In this study, three national regulatory agencies (US Food and Drug Administration—FDA; JP Pharmaceuticals and Medical Devices Agency—PMDA; and KR Ministry of Food and Drug Safety—MFDS) are selected after setting the following scope: (1) Establish a clear regulatory agency to conduct regulatory efforts for CDx or other in vitro diagnostic devices, (2) have experience with CDx regulatory approval, and (3) have at least 5 years of guideline development experience since the publication of the first guidance document for CDx devices.

### 2.2. Data Collection and Extraction

CDx approval data were analyzed by referring to publicly available approval reports in the databases of each regulatory agency [21,22,23,24,25,26,27,28,29]. The CDx data were divided according to the three technological principles (FISH, PCR, and NGS), and one of the cases with the most items evaluating analytical performance was selected. Different products were selected for each country to ensure the diversity of the device. Data on the safety and effectiveness of the device were extracted from the selected data, and analysis methods and results were analyzed according to the evaluation items.

**Figure 2:**
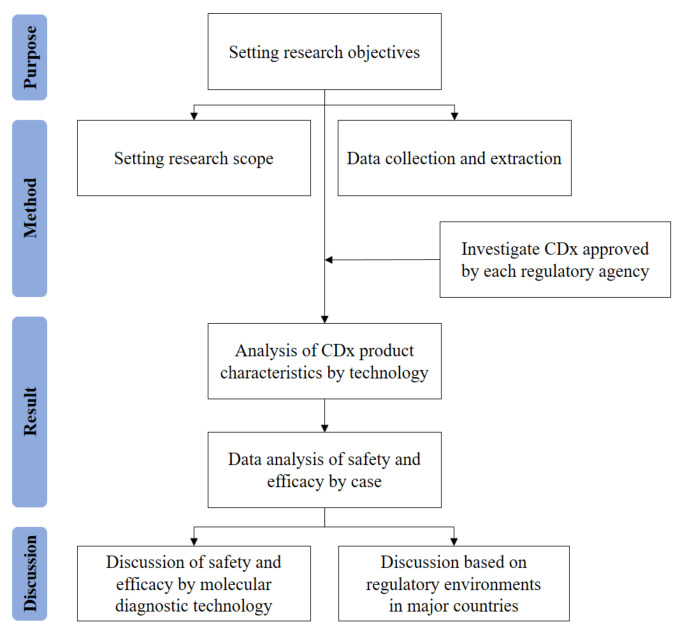
Flow chart of the research model.

In general, data that could confirm clinical effectiveness were analyzed based on performance (analytical and clinical performance tests) to evaluate the safety and effectiveness of in vitro diagnostic medical devices. In particular, due to the nature of CDx products, which require clinical efficacy or usefulness proven by clinical trial data, clinical drug trial data to which CDx testing was applied were also analyzed, where appropriate. The overall research model is shown in Figure 2.

## 3. Results

The CDx products approved by major regulatory agencies (FDA, PMDA, and MFDS) were analyzed by classifying them according to the technology used (FISH, PCR, and NGS), and a total of nine cases were analyzed.

### 3.1. FISH

#### 3.1.1. Vysis CLL FISH Probe Kit Approved by the US FDA

The Vysis CLL FISH Probe Kit (P150041), approved by the US FDA on 11 April 2016, is a CDx test developed by Abbott to identify patients with cellular chronic lymphocytic leukemia (CLL) who could benefit from treatment with venetoclax (Venclexta). The device uses a FISH-based method that detects the LSI TP53 probe target (17p-) in peripheral blood samples from previously untreated CLL patients. Safety and efficacy data are largely divided into analytical performance and clinical performance items. Analytical performance included analytical sensitivity, analytical specificity, normal cutoff, precision, reproducibility, robustness, probe concentration optimization and limitations, stability, and data on microbial interference response. For clinical performance, data on study design (design, inclusion and exclusion criteria, follow-up schedule, and clinical endpoints) and population characteristics (population characteristics and sample size) were presented according to the clinical study. In particular, due to the characteristics of the FISH technology used in the Vysis kit, items such as the response to abnormal signal patterns, hybridization temperature and time, photostability, cell reaction conditions, and hybrid slide stability were evaluated. The clinical performance of Vysis was approved through a multicenter phase 2 clinical trial study (Supplementary Material Table S3).

#### 3.1.2. Vysis ALK Break Apart FISH Probe Kit Approved by the JP PMDA

The Vysis ALK Break Apart FISH Probe Kit (22400AMX00630000), approved by the PMDA, is a CDx product developed by Abbott, and it determines the therapeutic application of undifferentiated lymphoma kinase inhibitors (crizotinib, alectinib hydrochloride, and brigatinib). This is a FISH-based device that detects the ALK fusion gene and visualizes the nucleic acid sequence of a specific chromosome in a cell. The method used by the device involves the hybridization of a probe to the DNA region within the cell, visualizing it using a fluorescence microscope, and then detecting it. As the approval report or review report data were not provided separately for this product, the safety and efficacy data were collected by referring to the attached documents registered in the PMDA database. Related items were divided into performance and clinical significance. The performance evaluations included data on analytical sensitivity, analytical specificity, reproducibility, non-specific reactions, probe signal intensity, slide background, cross-reactivity, microbiological tests, stability items, and clinical significance. For significance, data from concordance tests using clinical samples, clinical trials, equivalence studies, and brigatinib phase III international joint trials were presented. Vysis analyzed items such as the response to abnormal signal patterns due to the nature of FISH technology, sample selection according to cell and chromosome division stage, staining status, hybridization temperature and time, photostability, cell reaction conditions, and hybrid slide stability. The clinical significance of Vysis was approved by proving it through a concordance test or equivalence test in a pharmaceutical clinical trial study (Supplementary Material Table S4).

#### 3.1.3. PATHVYSION HER-2 DNA Probe Kit Approved by the KR MFDS

The PATHVYSION HER-2 DNA Probe Kit (in vitro License No. 13-610), approved by the MFDS, is a CDx product imported from Dow BioMedica and is used to detect the amplification of the HER-2/neu gene in human breast cancer tissue using the FISH method. This is a reagent used in an in vitro diagnostic analyzer that helps determine which patients should be prescribed trastuzumab (Herceptin). A human breast cancer tissue sample fixed with formalin and embedded in paraffin is treated with a fluorescent substance to target the gene, and then the image of the fluorescently labeled chromosome is analyzed using a medical image analysis device to detect the amplification of the HER-2/neu gene. Safety and efficacy data are largely divided into analytical and clinical performance items. Analysis performance included data on hybridization reaction efficiency, analytical sensitivity/specificity, the reproducibility and repeatability of preclinical studies, control slide reproducibility, portability items, and clinical performance data. In terms of clinical performance, data on clinical trials of pharmaceuticals and the evaluation of consistency with the test methods used in the studies were presented. PATHVYSION’s analysis performance was judged based on the analysis of items, such as the presence or absence of hybridization reactions due to the characteristics of FISH technology, sample selection according to the cell division period, the degree of gene amplification, and portability, considering point-of-care testing technology. The clinical performance of PATHVYSION was approved by verifying the correlation between gene amplification in clinical drug trials and survival rates according to drug use and evaluating consistency with clinical trial assays (CTAs) (Supplementary Material Table S5).

### 3.2. PCR

#### 3.2.1. CRCdx RAS Mutation Detection Kit Approved by the US FDA

The CRCdx RAS Mutation Detection Kit (P220005), approved by the US FDA on 29 September 2023, is a CDx product developed by Entrogen that identifies colorectal cancer (CRC) patients who can benefit from treatment with panitumumab (Vectibix). This quantitative rtPCR device can detect 35 types of KRAS and NRAS exon 2, 3, and 4 somatic mutations in genomic DNA extracted from formalin-fixed and paraffin-embedded (FFPE) CRC tissue samples. The safety and efficacy data were largely divided into analytical and clinical performance items, where analytical performance included accuracy, analytical sensitivity (blank limit, detection limit, equivalence, DNA input range, minimum tumor content, number of curls, and DNA extraction method characteristics), specificity (cross-reactivity and interference response), precision, reproducibility, guard banding, cross-contamination, and stability. For clinical performance, data on the study design (design, inclusion and exclusion criteria, follow-up schedule, and clinical feasibility) and population characteristics (population characteristics and sample size) according to the clinical study were presented. For analysis performance, due to the nature of rT-PCR technology, items such as DNA reaction conditions and tumor content ratio, cross-reaction of primers and probes, and stability to temperature response during PCR were presented. The clinical performance of CRCdx is a follow-on CDx device with the same purpose as the Praxis Extended RAS Panel (P160038), approved on 29 June 2017, and approval was obtained through a consistency study with the same device (Supplementary Material Table S6).

#### 3.2.2. therascreen EGFR RGQ PCR Kit Approved by the JP PMDA

The therascreen EGFR RGQ PCR Kit (22300AMX01256000), approved by the PMDA, is a CDx product developed by Qiagen that detects epidermal growth factor receptor (EGFR) gene mutations in DNA samples extracted from cancer tissue. The device is based on rtPCR technology for treatment decisions for patients with non-small cell lung cancer (NSCLC). The device detects three types of exon 18, 19 types of exon 19 deletions, T790M and S768I in exon 20, and L858R and L861Q in exon 21 of the EGFR gene using rtPCR and the Scorpions-amplification refractory mutation system method. Since the approval report or review of the report data was not provided separately for this product, the safety and efficacy data were collected by referring to the attached documents registered in the PMDA database. Related items were largely divided into performance and clinical significance. The analytical performance included data on the control Ct value, minimum detection limit, cross-reactivity, accuracy, precision, reproducibility, correlation, and interference response, and clinical significance data on afatinib. Data from the maleate clinical study, gefitinib clinical study, and dacomitinib clinical study were presented. Due to the nature of PCR technology, the items evaluated for therascreen included the reference range of a control group according to DNA content, the response to interfering substances such as proteinase K, and minimum detection concentrations for DNA-added samples. The clinical significance of therascreen was proven through a retrospective test of a clinical drug trial study and a concordance test, and approval was granted (Supplementary Material Table S7).

#### 3.2.3. therascreen KRAS RGQ PCR Kit Approved by the KR MFDS

The therascreen KRAS RGQ PCR kit (in Vitro License No. 19-164), approved by the MFDS, is a CDx product developed by Qiagen Korea. This is an in vitro diagnostic reagent that is used in rtPCR to detect seven somatic mutations in the human KRAS gene (G12A, G12D, G12R, G12C, G12S, and G12V in codon 12, and G13D in codon 13) in patients with CRC. It can be used to select patients for cetuximab (Erbitux^®^) treatment for rtPCR analysis to detect the G12C mutation in the human KRAS gene in DNA extracted from the FFPE lung tissue of patients with NSCLC. The device also uses rtPCR to help doctors select patients eligible for Sotorasib (Lumakras™) treatment. Safety and efficacy data were largely divided into analytical and clinical performance items. Analysis performance included cutoff, blank limit, detection limit, comparison with standard methods, the influence of input DNA, linearity and amplification efficiency, interfering substances, and cross-contamination. Data on items such as response, repeatability, and reproducibility were presented, and for clinical performance, data from a bridging study conducted using analyzable clinical samples from clinical drug trials were presented. Due to the nature of PCR technology, therascreen’s analytical performance evaluated items such as standards of detection concentration according to cutoffs, frequency, and gene content, as well as correlations with standard materials. The clinical performance of therascreen was approved through a bridging study with clinical drug trials using clinical samples (Supplementary Material Table S8).

### 3.3. NGS

#### 3.3.1. Praxis Extended RAS Panel Approved by the US FDA

The Praxis Extended RAS Panel (P160038), approved by the US FDA on 29 June 2017, is a CDx product developed by Illumina that identifies CRC patients who can benefit from treatment with panitumumab (Vectibix). Praxis is an NGS-based CDx product that detects 56 mutations in exons 2, 3, and 4 of the KRAS and NRAS genes using the Illumina MiSeqDx instrument in CRC FFPE tissue samples. Praxis was approved by determining its safety and effectiveness compared to Sanger sequencing, which was used as the reference method. Safety and efficacy data were largely divided into analytical and clinical performance items. Analytical performance evaluations include accuracy, analytical sensitivity (blank limit, detection limit, and DNA input), analytical specificity (interference response to exogenous and endogenous substances), precision, reproducibility, equivalence, PCR thermocycler system comparison, quantitative PCR system comparison, sample carryover, specimen handling, guard banding, and stability items. Clinical performance evaluations include study design (inclusion and exclusion criteria, follow-up schedules, and clinical endpoints) and population characteristics (population characteristics, sampling criteria, and sample size). Due to the nature of NGS technology, Praxis analyzed items such as DNA input and the tumor content ratio. Additional comparisons with PCR systems used when conducting research and guard banding at each stage (sample eligibility, library preparation, and sequencing) were made. For clinical performance, a retrospective study was conducted using samples collected and stored in an existing Amgen clinical trial, and the rate of survival improvement due to the drug, which is a clinical benefit, was evaluated (Supplementary Material Table S9).

#### 3.3.2. OncoGuide NCC Oncopanel System Approved by JP PMDA

The OncoGuide NCC Oncopanel System, approved by the PMDA on 28 June 2018, is a CDx product developed by Sysmax and consists of a template DNA extraction reagent and a software analysis program. It is an NGS-based device that provides information on genetic mutations to help establish treatment plans based on the comprehensive genomic profiling of 114 cancer-related genes collected from patients with solid tumors. The safety and efficacy data were largely divided into analytical performance and clinical performance items. Analytical performance included data on accuracy, precision, detection limit, tissue type, specificity, interference response, and comparison with the prototype. Clinical performance included data on the appropriateness of the target gene, the appropriateness of sensitivity for detecting target mutations, and the appropriateness of result report writing and content. Due to the nature of NGS technology, NCC analyzed items such as the base substitution of mutations, the accuracy of detecting indels, the frequency of alleles, and comparison with other analysis methods to verify the gene detection rate. In addition, clinical performance was evaluated based on the appropriateness of the proposed target gene, the appropriateness of sensitivity for detecting target mutations, and the appropriateness of result report writing and content rather than additional clinical research data. Ultimately, the product was approved based on the device’s intended use and clinical benefits (Supplementary Material Table S10).

#### 3.3.3. Oncomine Dx Target Test Approved by the KR MFDS

The Oncomine Dx Target Test (in vitro License No. 18-370), approved by the MFDS, is an NGS-based CDx imported from Thermo Fisher Scientific. The CDx detects the following genes: single-nucleotide variations (SNVs), deletions and insertions in 23 genes in DNA isolated from FFPE tissue samples obtained from NSCLC patients with RET and ROS1 fusion in RNA, single-nucleotide sequence mutations, multiple-nucleotide sequence mutations, and deletion of the RET gene in DNA isolated from FFPE tissue samples obtained from medullary thyroid cancer patients with RET fusion in RNA isolated from FFPE tissue samples obtained from thyroid cancer patients. The safety and efficacy data were largely divided into analytical and clinical performance items. Analysis performance included blank limits, tissue content, guard banding, stability, DNA/RNA input, cross-reactions, cross-contamination, tissue fixation, reproducibility, interfering reactions, and minimum data on the detection limit, tumor cell content, repeatability, and accuracy items. For clinical performance, data on the evaluation of the agreement rate between control and test groups using clinical samples for each biomarker were presented. Due to the nature of NGS technology, Oncomine’s performance was evaluated using items such as tissue and tumor cell content, library storage conditions, DNA/RNA input, and the minimum detection limit according to the workflow for each biomarker. Clinical performance was approved through a concordance test with standard methods using clinical samples for each biomarker, and clinical effectiveness was verified using data from clinical drug trials. For clinical performance, only data for NSCLC were summarized because the data elements were identical to those for thyroid cancer (Supplementary Material Table S11).

### 3.4. Comparison of Differences in Safety and Efficacy Assessments of Diagnostic Technology

After examining cases from each major regulatory agency, common items were identified. These included accuracy, precision, reproducibility, specificity, sensitivity, detection limits, analysis cutoff, interfering reactions, cross-reaction, and stability. However, differences appeared in the characteristics evaluated according to the principles of each technology (Table 1).

## 4. Discussion

This review analyzed CDx products using molecular diagnostic technologies (FISH, PCR, and NGS) approved by regulatory agencies to discuss their safety and effectiveness, focusing on the diagnostic field of precision medicine.

The study findings confirmed that the safety and effectiveness requirements of CDx differed depending on which molecular diagnostic technology was applied. In terms of the analysis procedures, FISH had to ensure performance in terms of staining intensity and time, and an analysis of probe concentrations and photostability was necessary. The efficiency and quality of hybridization can have a huge impact on diagnostic results; therefore, conditions for variables related to culture pretreatment and 4′,6-diamidino-2-phenylindole binding, which generate non-specific signals, must be optimized [30]. The Vysis CLL FISH Probe Kit showed a high overall response rate (80.2%; 95% confidence interval (CI) 71–87%) in patients with chromosome 17p deletion who had received at least one CLL treatment. Through this clinical trial, the efficacy of Venetoclax, the first BCL-2 inhibitor, was proven and approved by the FDA [31]. In addition, the 4-year follow-up of the Vysis kit using molecular biomarker analysis still showed high progression-free survival (PFS) and overall survival (OS) rates, indicating its long-term genetic value [32]. The Vysis ALK Break Apart FISH Probe Kit showed a high success rate (83%) for ALK rearrangement in the ALK-positive cohort despite the lengthy transport distance (slides were transported between Korea and Singapore) [33], and subsequently, many studies have reported its safety and effectiveness [34,35,36]. The technical and economic value of the PATHVYSION HER-2 DNA Probe Kit has been recognized by demonstrating better sensitivity and lower background staining than existing devices [37]. The reliability of the technology used by this device was verified by a high agreement rate (98%) in a large-scale comparative study with the latest FISH kit [38].

PCR evaluation requires information on DNA input, minimum tumor content, and the specificity of primers and probes. Because PCR is based on the principle of amplifying and detecting DNA, the purity and quality of DNA can affect the sensitivity to mutations, and retesting may be necessary due to discrepancies [39]. Mutation testing using the therascreen EGFR RGQ PCR Kit involves detecting target DNA through a six-step extraction process: deparaffinization, lysis using proteinase K, incubation at 90 °C for cross-linking reversal, DNA–membrane binding, removing residual contamination, and DNA elution. Dissolution conditions and culture temperature must be adjusted to improve the yield and performance of DNA, and optimal conditions for the efficient detection of DNA must be considered [40]. The clinical usefulness of this kit was proven by performance studies, such as comparisons of agreement with reference methods and various clinical practice studies [41]. The therascreen KRAS RGQ PCR kit demonstrated excellent performance in a comparison of the workflow of three commercially available KRAS mutation detection platforms [42]. The detection of KRAS mutations in clinical samples requires consideration of the quality of the DNA, as it depends on the technical performance of detecting mutant alleles in normal wild-type alleles, template degradation to detect target DNA, and well-defined thresholds and signal strength [43].

NGS was characterized by comparison with PCR and guard banding items at each workflow stage (sample qualification, library preparation, and sequencing). The FDA appeared to evaluate items related to analytical performance the most. This suggests that a huge amount of analytical data may be required because the FDA expects most non-US companies to conduct research at at least three different sites [44]. The Praxis Extended RAS Panel replaces PCR technology, which lacks the ability to detect and simultaneously screen for multiple genetic alterations, improving diagnostic yield with only small DNA input [45]. It has been recognized for its clinical value by demonstrating thorough pathological evaluations, high concordance rates on Sanger sequencing, and prolonged PFS and OS [46]. Additional studies have shown that the double-stranded approach removes many interfering substances that may be generated in FFPE tissue [47]. The OncoGuide NCC Oncopanel System was shown to have high analytical sensitivity and clinical effectiveness, and the proportion of samples most optimized for the device was analyzed to evaluate whether it met appropriate standards [48,49]. However, tumor heterogeneity, sample type, and the analytical characteristics of each panel, which may lead to analytical inconsistencies between devices, may also need to be considered [50]. The Oncomine Dx Target Test has been the most evaluated in recent studies for its feasibility in mutation screening [51,52,53,54,55]. Studies that excluded existing performance comparisons suggested that it is important to derive factors that can affect the test success rate of gene detection and considerations that are relevant to treatment decisions [56,57,58,59]. In common clinical performance, when using clinical drug trial data, improvements in patient PFS, objective response rate (ORR), and OS must be confirmed, and when comparing or evaluating equivalence with existing methods, concordances such as overall percent agreement, positive percent agreement, and negative percent agreement must be evaluated. Thus, the research evaluations required to prove the safety and effectiveness of a device may vary depending on the technology applied to the device and its principles [60].

Regulatory requirements for the safety and effectiveness of CDx devices have a common factor: they may require clinical drug trial data to ensure accuracy and reliability depending on the proposed use to ensure clinical effectiveness or usefulness, which can be demonstrated by analytical and clinical performances. However, since safety and efficacy verification requirements differ by the regulatory authority, the characteristics of each regulatory agency may need to be considered. The FDA places emphasis on the correlation between a device’s output and its intended use and clinical reference standards. This is similar to requiring a method comparison study with an already licensed product or technology according to the guidance document and suggests that it is consistent with a study that is expected to be conducted based on standardized performance studies, such as the Clinical & Laboratory Standards Institute [44]. Analytical performance must be proven by collecting and maintaining samples appropriate for the patient’s pathological characteristics, and clinical performance must include the ability to predict treatment results for individual patients and collect clinical trial data for pharmaceuticals. Additionally, if a device that is scheduled for final commercial release cannot be used in research, prototype testing can be performed, or a bridging study that demonstrates similarity to existing research (CTA) can be performed [60,61]. The PMDA evaluates the intended use and impact on safety and efficacy according to clinical significance. In particular, whether the detection capabilities of the device are consistent before and after the intended change in conditions should be described. If the device is not used in confirmatory clinical trials of pharmaceuticals, consistency should be evaluated under the same conditions as clinical trials. In principle, clinical performance should be proven through prospective randomized controlled trials of confirmatory clinical pharmaceutical trials; however, retrospective studies are also possible in some cases [62]. The PMDA’s guidelines, unlike other regulatory agencies, emphasize the need to include biomarker-negative patients in early clinical trials. This suggests that potential safety and efficacy should be judged by analyzing differences in the risk–benefit balance between biomarker-positive and -negative patients [63]. The MFDS evaluates according to the proposed use of the device, the rationality of safety and effectiveness, and the risk level [64]. This emphasizes that since the performance of the device directly affects the safety and effectiveness of the treatment prescription, a risk factor-based approach must be applied to evaluate the risk of false-positive and false-negative results [65,66]. Research methods must demonstrate consistency with existing test methods and confirm drug reactivity using CDx devices. They can also be used to evaluate equivalence with existing products [67].

This review provides safety and efficacy information according to the type of molecular diagnostic technology for regulatory considerations of in vitro diagnostics approval as CDx products in the US, Japan, and Korea. However, sufficient data were not collected because one case was analyzed for each regulatory agency for each molecular diagnostic technology. Therefore, the regulations cannot be applied or generalized to all applicable CDx products. Nonetheless, these individual case studies may be important from a regulatory perspective because CDx products must be evaluated, and analytical and clinical performance and usability must be documented for regulatory approval. In conclusion, the objective validity of this study was demonstrated by relying on actual approval case data rather than a comprehensive registry and analyzing information on product-specific and clinical performance evaluations in detail.

## 5. Conclusions

In precision medicine, molecular diagnostic technology is applied to CDx testing for personalized treatment to quickly determine the etiology of complex diseases using a minimum number of samples. The CDx safety and effectiveness requirements differ depending on which molecular diagnostic technology is applied. In particular, the preprocessing method for molecular diagnostic technology, pathological evaluation method, sample type, and the influence of interfering substances play an important role in the performance of CDx products. These regulatory discussions could be an approach that leads to the approval of CDx products that are appropriate for each country’s regulatory environment.

## Figures and Tables

**Table 1:** Differences in safety and efficacy assessments by diagnostic technique.

	Analytical Performance	Clinical Performance
FISH	Probe concentration optimization and limits Photostability Peripheral blood hybridization success rate Specimen and cell pellet, hybrid stability Probe signal strength	EOCG evaluation Statistical correlation evaluation using Cox proportional hazards model Equivalence test with standard methods Evaluation of agreement rate with CTA results
PCR	DNA input Minimum tumor content Curl count characteristics of DNA extraction method Guard banding	Bridge research with pharmaceutical clinical trials Evaluation of agreement rate with CTA results Proof of non-inferiority Setting standards for tumor area and tumor content Wilcoxon signed sequence test False-positive and false-negative results
NGS	DNA input PCR comparison Guard banding Tissue content Tumor cell content Sample carryover	Evaluation of consistency with existing products Accuracy evaluation of the control group Bridge research with CTA results Evaluation of agreement rate with LLT results Proof of reasons for inconsistent samples Panel-based subtype classification

Abbreviations: EOCG: Eastern Cooperative Oncology Group; CTA: clinical trial assay; DNA: deoxyribonucleic acid; PCR: polymerase chain reaction; LLT: local laboratory test.

## Data Availability

Data are available from the studies included in the review that have been cited.

## Supplementary Materials

The following supporting information can be downloaded at: https://www.mdpi.com/article/10.3390/life14111358/s1, Table S1: CDx guideline development status by the regulatory authority; Table S2: Status of CDx products approved by regulatory agencies; Table S3: Summary of safety and effectiveness of the Vysis CLL FISH Probe Kit approved by the US FDA; Table S4: Summary of safety and effectiveness of the Vysis ALK Break Apart FISH Probe Kit approved in JP PMDA; Table S5: Summary of safety and effectiveness of the PATHVYSION HER-2 DNA Probe Kit approved in KR MFDS; Table S6: Summary of safety and effectiveness of the CRCdx RAS Mutation Detection Kit approved by the US FDA; Table S7: Summary of safety and effectiveness of the therascreen EGFR RGQ PCR Kit approved in JP PMDA; Table S8: Summary of safety and efficacy of the therascreen KRAS RGQ PCR Kit approved in KR MFDS; Table S9: Summary of safety and effectiveness of the Praxis Extended RAS Panel approved by the US FDA; Table S10: Summary of safety and effectiveness of the OncoGuide NCC Oncopanel System approved in JP PMDA; Table S11: Summary of safety and effectiveness of the Oncomine Dx Target Test approved in KR MFDS.
