# Supplementary material for: Companion Diagnostics (CDx) Based on Molecular Biology Techniques
Source: Life (Basel). 2024 Oct 23;14(11):1358. doi: 10.3390/life14111358 (PMC11595734; doi:10.3390/life14111358)
Supplement: Supplementary file 1 [file life-14-01358-s001.zip › life-3218945-supplementary.pdf]

Supplemental material

Table S1. CDx guideline development status by the regulatory authority

Country	Regulatory authority	Number of approvals	CDx guideline development status
USA	US Food and Drug Administration (FDA)	57	1) In Vitro Companion Diagnostic Devices[2014.08]<br>2) Principles for Codevelopment of and In Vitro companion Diagnostic Device with a Therapeutic Product[2016.07]<br>3) Developing and Labeling In Vitro Companion Diagnostic Devices for a Specific Group of Oncology Therapeutic Products[2020.04]<br>4) U.S. Food & Drug Administration. Procedures for Handling Post-Approval Studies Imposed by Premarket Approval Application Order[2022.10]<br>5) Oncology Drug Products Used with Certain In Vitro Diagnostic Tests: Pilot Program[2023.06]
Europe	European Medicines Agency (EMA)	-	REGULATION (EU) 2017/746 OF THE EUROPEAN PARLIAMENT AND OF THE COUNCIL of 5 April 2017 on in vitro diagnostic medical devices and repealing Directive 98/79/EC and Commission Decision 2010/227/EU[2017.5]
Australia	Therapeutic Goods Administration (TGA)	≥17	IVD companion diagnostics Guidance on regulatory requirements[2022.10]
Japan	Pharmaceutical and Medical Devices Agency (PMDA)	43	1) Notification on Approval Application for In Vitro Companion Diagnostics and Corresponding Therapeutic Products[2013.07]<br>2) Technical Guidance on Development of In Vitro Companion Diagnostics and Corresponding Therapeutic Products[2013.12]<br>3) Notification on Handling of In Vitro Diagnostics and Medical Device Products Aiming for Drug-agnostic Companion Diagnostics[2022.03]<br>4) [2022.07]
China	National Medical Products Administration (NMPA)	-	1) Revision for instructions for use and technical review of tumor companion diagnostics based on similar therapeutic drugs(Draft)[2020.07]<br>2) Technical Review of CDx for the Same Kind of Therapeutic Drug[2020.07]<br>3) Guidance for Clinical Study of CDx for the Marketed Oncology Drugs[2020.08]<br>4) Guidelines for Technical Review and IFU Updates of Tumor Companion Diagnostics Reagents based on Similar Treatment Drugs[2021.04]<br>5) Guidelines for Registration Review of Original Companion Diagnostics Reagents Co-developed (Simultaneously) with Anti-Tumor Drugs[2022.06]
Korea	Ministry of Food and Drug Safety (MFDS)	30	1) In vitro companion diagnostic device approval and review guidelines (civilian guide) [2015.10]<br>2) Guidelines for safety/performance and clinical trial protocol evaluation of companion diagnostic medical devices using liquid biopsy (Civilian guide) [2018.02]<br>3) Explanation on significance verification of NGS-based genetic testing (Civilian guide) [October 2022]<br>4) Companion diagnostic medical device approval and review guidelines (Civilian guide) [2022.12]

** As of December 26, 2023*
** The number of permits is the result of a search from the database of each regulatory agency, and cases for which permit information cannot be obtained due to public transparency are marked (-) separately.*

Table S2. Status of CDx products approved by regulatory agencies

№	Product Name	Manufacturer	Disease	Diagnosis Principle	Gene/Biomarker
**FDA**					
1	AAV5 DetectCDx	ARUP Laboratories	Hemophilia A	Electrochemiluminescence	*AAV5*
2	Abbott RealTime IDH1	Abbott Molecular, Inc.	Acute myeloid leukemia	PCR	*IDH1*
3	Abbott RealTime IDH2	Abbott Molecular, Inc.	Acute myeloid leukemia	PCR	*IDH2*
4	Agilent RESOLUTION CtDx FIRST	Resolution Bioscience, Inc.	Non-small cell lung cancer	NGS	*KRAS*
5	Bond Oracle HER2 IHC System	Leica Biosystems	Breast cancer	IHC	*HER2*
6	BRACAnalysis CDx™	Myriad Genetic Laboratories, Inc.	Ovarian cancer, Breast cancer, Pancreatic cancer, Metastatic castration-resistant prostate cancer	PCR, Sanger Sequencing	*BRCA1/2*
7	cobas 4800 BRAF V600 Mutation Test	Roche Molecular Systems, Inc.	Melanoma	Real-Time PCR	*BRAF*
8	cobas EGFR mutation test v1	Roche Molecular Systems, Inc.	Non-small cell lung cancer	Real-Time PCR	*EGFR*
9	cobas EGFR mutation test v2	Roche Molecular Systems, Inc.	Non-small cell lung cancer	Real-Time PCR	*EGFR*
10	cobas EZH2 Mutation Test	Roche Molecular Systems, Inc.	Follicular lymphoma tumor	Real-Time AS-PCR	*EZH2*
11	cobas KRAS Mutation Test	Roche Molecular Systems, Inc.	Colorectal cancer	Real-Time PCR	*KRAS*
12	CRCDx RAS Mutation Detection Assay Kit	EntroGen, Inc.	Colorectal cancer	Real-Time PCR	*RAS*
13	Dako c-KIT pharmDx	Dako North America, Inc.	Gastrointestinal stromal tumor	IHC	*C-KIT PROTEIN/CD117 ANTIGEN*
14	Dako EGFR pharmDx Kit	Dako North America, Inc.	Colorectal cancer	IHC	*EGFR*
15	FerriScan	Resonance Health Analysis Services Pty Ltd	Non-transfusion dependent thalassemia	MRI	*Imaging*
16	FoundationFocus CDxBRCA Assay	Foundation Medicine, Inc.	Ovarian cancer	NGS	*BRCA1/2*
17	FoundationOne CDx	Foundation Medicine, Inc.	Ovarian cancer, Non-small cell lung cancer, Breast cancer, Colorectal cancer, Melanoma, Cholangiocarcinoma, Metastatic castration-resistant prostate cancer, Solid tumor	NGS	*MSI*

18	FoundationOne Liquid CDx	Foundation Medicine, Inc.	Non-small cell lung cancer, Metastatic castration-resistant prostate cancer, Solid tumor, Ovarian cancer, Breast cancer	NGS	*EGFR*
19	Guardant360 CDx	Guardant Health, Inc.	Non-small cell lung cancer, Breast cancer	NGS	*KRAS G12C*
20	HER2 CISH pharmDx Kit	Dako Denmark A/S	Breast cancer	CISH	*HER2*
21	HER2 FISH pharmDx Kit	Dako Denmark A/S	Breast cancer, Stomach cancer and gastroesophageal cancer	FISH	*HER2*
22	HercepTest	Dako Denmark A/S	Breast cancer, Stomach cancer and gastroesophageal cancer	IHC	*HER2*
23	INFORM HER2 Dual ISH DNA Probe Cocktail	Ventana Medical Systems, Inc.	Breast cancer	CISH	*HER2*
24	INFORM HER-2/neu	Ventana Medical Systems, Inc.	Breast cancer	FISH	*HER2*
25	InSite Her-2/neu KIT	Biogenex Laboratories, Inc.	Breast cancer	IHC	*HER2*
26	Ki-67 IHC MIB-1 pharmDx	Agilent Technologies	Breast cancer	IHC	*Ki-67*
27	KIT D816V Mutation Detection by PCR for Gleevec Eligibility in Aggressive Systemic Mastocytosis (ASM)	ARUP Laboratories, Inc.	Aggressive systemic mastocytosis	PCR	*KIT D816V*
28	LeukoStrat CDx FLT3 Mutation Assay	Invivoscribe Technologies, Inc.	Acute myeloid leukemia	PCR	*FLT3*
29	MRDx BCR-ABL test	MolecularMD Corporation	Chronic myeloid leukemia	Real-Time PCR	*BCR-ABL1*
30	Myriad myChoice CDx	Myriad Genetic Laboratories, Inc.	Ovarian cancer	NGS	*BRCA1/2*
31	ONCO/Reveal Dx Lung & Colon Cancer Assay(O/RDx-LCCA)	Pillar Biosciences, Inc.	Colorectal cancer, Non-small cell lung cancer	NGS	*KRAS, EGFR*
32	Oncomine Dx Target Test	Life Technologies Corporation	Non-small cell lung cancer, Cholangiocarcinoma, Medullary thyroid cancer, Thyroid cancer	NGS	*BRAF, EGFR, IDH1, ROS1, RET*
33	PathVysion HER-2 DNA Probe Kit	Abbott Molecular Inc.	Breast cancer	FISH	*HER2*
34	PATHWAY anti-Her2/neu(4B5) Rabbit Monoclonal Primary Antibody	Ventana Medical Systems, Inc.	Breast cancer	IHC	*HER2*

35	PD-L1 IHC 22C3 pharmDx	Dako North America, Inc.	Non-small cell lung cancer, Cervical cancer, Head and neck squamous cell carcinoma, Esophageal squamous cell carcinoma, Triple-negative breast cancer	IHC	*PD-L1*
36	PD-L1 IHC 28-8 pharmDx	Dako North America, Inc.	Non-small cell lung cancer	IHC	*PD-L1*
37	PDGFRB FISH Assay	ARUP Laboratories, Inc.	Myelodysplastic syndrome/ myeloproliferative disease	FISH	*PDGFRB*
38	POMC/PCSK1/LEPR CDx Panel	PreventionGenetics, LLC	Obesity	NGS	*POMC, PCSK1, LEPR*
39	Praxis Extended RAS Panel	Illumina, Inc.	Colorectal cancer	NGS	*RAS*
40	SeCore CDx HLA Sequencing System	One Lambda Inc.	Uveal melanoma	Sanger Sequencing	*HLA A*02:01*
41	SPOT-LIGHT HER2 CISH Kit	Life Technologies Corporation	Breast cancer	CISH	*HER2*
42	therascreen BRAF V600E RGQ PCR Kit	QIAGEN GmbH	Colorectal cancer	Real-Time PCR	*BRAF*
43	therascreen EGFR RGQ PCR Kit	Qiagen Manchester, Ltd.	Non-small cell lung cancer	Real-Time PCR	*EGFR*
44	therascreen FGFR RGQ RT-PCR Kit	Qiagen Manchester, Ltd.	Urothelial cancer	Real-Time PCR	*FGFR*
45	therascreen KRAS RGQ PCR Kit	Qiagen Manchester, Ltd.	Colorectal cancer, Non-small cell lung cancer	Real-Time PCR	*KRAS*
46	therascreen PIK3CA RGQ PCR Kit	QIAGEN GmbH	Breast cancer	Real-Time PCR	*PIK3CA*
47	therascreen PDGFRA RGQ PCR Kit	QIAGEN GmbH	Gastrointestinal stromal tumor	Real-Time PCR	*PDGFRA*
48	THXID BRAF Kit	bioMérieux Inc.	Melanoma	Real-Time PCR	*BRAF*
49	VENTANA ALK(D5F3) CDx Assay	Ventana Medical Systems, Inc.	Non-small cell lung cancer	IHC	*ALK*
50	VENTANA FOLR1 (FOLR-2.1) RxDx Assay	Ventana Medical Systems, Inc.	Epithelial ovarian cancer, Fallopian tube cancer, or primary peritoneal cancer	IHC	*FOLR1*
51	VENTANA HER2 Dual ISH DNA Probe Cocktail	Ventana Medical Systems, Inc.	Breast cancer	CISH	*HER2*
52	VENTANA MMR RxDx Panel	Ventana Medical Systems, Inc.	Endometrial carcinoma, solid tumor	IHC	*MMR, MLH1, PMS2, MSH2, MSH6*

53	VENTANA PD-L1(SP142) Assay	Ventana Medical Systems, Inc.	Urothelial carcinoma, Non-small cell lung cancer	IHC	*PD-L1*
54	VENTANA PD-L1(SP263) Assay	Ventana Medical Systems, Inc.	Non-small cell lung cancer	IHC	*PD-L1*
55	Vysis ALK Break Apart FISH Probe Kit	Abbott Molecular Inc.	Non-small cell lung cancer	FISH	*ALK*
56	Vysis CLL FISH Probe Kit	Abbott Molecular Inc.	B-cell chronic lymphocytic leukemia	FISH	*LSI TP53, LSI ATM, LSI D13S319, D12Z3*
57	xT CDx	Tempus Labs, Inc.	Solid tumor	NGS	*KRAS, NRAS*
**PMDA**					
1	AmoyDx Pan Lung Cancer PCR Panel	Amoy Diagnostics	Non-small cell lung cancer	Real-Time PCR	*EGFR, ALK, ROS1, KRAS, BRAF, HER2, RET, MET, NTRK1, NTRK2, NTRK3*
2	BRACAnalysis CDx	Myriad Genetic Laboratories, Inc.	Ovarian cancer, Breast cancer, Pancreatic cancer, Metastatic castration-resistant prostate cancer	PCR, Sanger Sequencing	*BRCA1/2*
3	cobas BRAF V600 mutation test	Roche Molecular Systems, Inc.	Melanoma	Real-Time PCR	*BRAF*
4	cobas EGFR mutation test v2	Roche Molecular Systems, Inc.	Non-small cell lung cancer	Real-Time PCR	*EGFR*
5	cobas EZH2 Mutation Test	Roche Molecular Systems, Inc.	Follicular lymphoma tumor	Real-Time AS-PCR	*EZH2*
6	EGFR LIQUID	DNA Chip Research Inc	Non-small cell lung cancer	NGS	*EGFR*
7	FoundationOne CDx	Foundation Medicine, Inc.	Ovarian cancer, Non-small cell lung cancer, Breast cancer, colorectal cancer, Melanoma, Cholangiocarcinoma, Metastatic castration-resistant prostate cancer, Solid tumor	NGS	*MSI*
8	FoundationOne Liquid CDx	Foundation Medicine, Inc.	Non-small cell lung cancer, Metastatic castration-resistant prostate cancer, Solid tumor, Ovarian cancer, Breast cancer	NGS	*EGFR*
9	Guardant360 CDx	Guardant Health, Inc.	Non-small cell lung cancer, Breast cancer	NGS	*KRAS G12C*
10	Histofine ALK iAEP kit	Nichirei Bioscience	Non-small cell lung cancer	IHC	*ALK*

11	Histra HER2 FISH Kit	JOKOH	Breast cancer	FISH	*HER2*
12	Idylla MSI Test	BIOCARTIS	Colorectal cancer	PCR	*MSI*
13	Idylla RAS-BRAF Mutation Test	BIOCARTIS	Colorectal cancer	Real-Time PCR	*KRAS, NRAS, BRAF*
14	LeukoStrat CDx FLT3 Mutation Assay	invivoscribe	Acute myeloid leukemia	PCR	*FLT3*
15	Lung Cancer Compact Panel Dx Multiplex Companion Diagnostic System	DNA Chip Research Inc	Non-small cell lung cancer	NGS	*EGFR, ALK, ROS1, MET, BRAF, RET, NTRK*
16	MEBCDX AAV9 TEST	JSR Life Sciences	Spinal muscular atrophy	ELISA	*AAV9*
17	MEBGEN BRAF 3 Kit	JSR Life Sciences	Melanoma	PCR	*BRAF*
18	MEBGEN BRAF Kit	JSR Life Sciences	Solid tumor, Hairy cell leukemia	PCR	*BRAF*
19	MEBGEN RASKET-B kit	JSR Life Sciences	Colorectal cancer	PCR	*RAS, BRAF*
20	MSI kit	BIORAD	Solid tumor, Colorectal cancer	ddPCR	*BAT25, BAT26, NR21, NR24, Mono27*
21	myChoice CDx	Myriad Genetic Laboratories, Inc.	Ovarian cancer	NGS	*BRCA1/2*
22	OncoBEAM RAS CRC Kit	Sysmex	Colorectal cancer	ddPCR	*RAS*
23	OncoGuide AmoyDx ROS1 Gene Fusions Detection Kit	Amoy Diagnostics	Non-small cell lung cancer	Real-Time PCR	*ROS1*
24	OncoGuide NCC Oncopanel System	Sysmex	Solid tumor	NGS	*FGFR2*
25	Oncomine Dx Target Test	ThermoFisher SCIENTIFIC	Non-small cell lung cancer	NGS	*BRAF, EGFR, IDH1, ROS1, RET*
26	PathVysion HER-2 DNA Probe Kit	Abbott Molecular Inc.	Breast cancer	FISH	*HER2*
27	PD-L1 IHC 22C3 pharmDx	Dako North America, Inc.	Non-small cell lung cancer, Cervical cancer, Head and neck squamous cell carcinoma, Esophageal squamous cell carcinoma, Triple-negative breast cancer	IHC	*PD-L1*
28	POTELIGEO TEST FCM	Kyowa Medex	Adult T-cell leukemia lymphoma	Flow cytometry	*CCR4*
29	POTELIGEO TEST IHC	Kyowa Medex	Adult T-cell leukemia lymphoma	IHC	*CCR4*

30	therascreen BRAF V600E RGQ PCR Kit	QIAGEN	Colorectal cancer	Real-Time PCR	*BRAF*
31	therascreen EGFR RGQ PCR kit	QIAGEN	Non-small cell lung cancer	Real-Time PCR	*EGFR*
32	therascreen KRAS RGQ PCR Kit	QIAGEN	Colorectal cancer, Non-small cell lung cancer	Real-Time PCR	*KRAS*
33	THxID BRAF kit	bioMérieux Inc.	Melanoma	Real-Time PCR	*BRAF*
34	VENTANA DISH HER2 kit	Ventana Medical Systems, Inc.	Salivary gland tumor	CISH	*HER2*
35	Ventana OptiView ALK (D5F3)	Ventana Medical Systems, Inc.	Non-small cell lung cancer	IHC	*ALK*
36	VENTANA OptiView MLH1（M1）	Ventana Medical Systems, Inc.	Colorectal cancer	IHC	*MLH1*
37	VENTANA OptiView MSH2（G219-1129）	Ventana Medical Systems, Inc.	Colorectal cancer	IHC	*MSH2*
38	VENTANA OptiView MSH6（SP93）	Ventana Medical Systems, Inc.	Colorectal cancer	IHC	*MSH6*
39	VENTANA OptiView PD-L1（SP142）	Ventana Medical Systems, Inc.	Urothelial carcinoma, Non-small cell lung cancer	IHC	*PD-L1*
40	VENTANA OptiView PD-L1（SP263）	Ventana Medical Systems, Inc.	Non-small cell lung cancer	IHC	*PD-L1*
41	VENTANA OptiView PMS2（A16-4）	Ventana Medical Systems, Inc.	Colorectal cancer	IHC	*PMS2*
42	Ventana ultraView Pathway HER2 (4B5)	Ventana Medical Systems, Inc	Breast cancer	IHC	*HER2*
43	Vysis ALK Break Apart FISH probe kit	Abbott Molecular Inc.	Non-small cell lung cancer	FISH	*ALK*
**MFDS**					
1	AmoyDx ROS1 Gene Fusions Detection Kit	Clinomics	Non-small cell lung cancer	Real-Time PCR	*ROS1*
2	cobas 4800 BRAF V600 Mutation Test	Roche Diagnostics	Papillary thyroid cancer, Melanoma	PCR	*BRAF*
3	cobas EGFR Mutation Test v2	Roche Diagnostics	Non-small cell lung cancer	Real-Time PCR	*EGFR*
4	Droplex BRAF Mutation Test v2	Gencurix	Papillary thyroid cancer, Melanoma	ddPCR	*BRAF*
5	Droplex EGFR Mutation Test v2	Gencurix	Non-small cell lung cancer	ddPCR	*EGFR*
6	Droplex KRAS Mutation Test	Gencurix	Rectal cancer	ddPCR	*KRAS*
7	Droplex KRAS Mutation Test v2	Gencurix	Colorectal cancer	ddPCR	*KRAS*
8	Droplex PIK3CA Mutation Test	Gencurix	Breast cancer	ddPCR	*PIK3CA*

9	GenesWell™ ddEGFR Mutation Test	Gencurix	Non-small cell lung cancer	ddPCR	*EGFR*
10	INFORM HER2 Dual ISH DNA Probe Cocktail	Roche Diagnostics	Breast cancer	ISH	*HER2*
11	Oncomine Dx Target Test and Controls Kit	Thermo Fisher Scientific	Non-small cell lung cancer, Medullary thyroid cancer, Thyroid cancer	NGS	*BRAF, EGFR, IDH1, ROS1, RET*
12	OncoTector™ KRAS Mutation Detection kit	HLB Panagene	Non-small cell lung cancer	Real-Time PCR	*KRAS*
13	PANAMutyper™ R EGFR	HLB Panagene	Non-small cell lung cancer	Real-Time PCR	*EGFR*
14	PANAMutyper™ R EGFR V2	HLB Panagene	Non-small cell lung cancer	Real-Time PCR	*EGFR*
15	PATHVYSION HER-2 DNA Probe Kit	Dowbiomedica	Breast cancer	FISH	*HER2*
16	PD-L1 IHC 22C3 pharmDx, GE006	Agilent Technologies	Non-small cell lung cancer	IHC	*PD-L1*
17	PD-L1 IHC 22C3 pharmDx, SK006	Agilent Technologies	Non-small cell lung cancer, Head and neck squamous cell cancer, Gastric adenocarcinoma, Gastroesophageal junction adenocarcinoma, Esophageal cancer	IHC	*PD-L1*
18	PD-L1 IHC 28-8 pharmDx, SK005	Agilent Technologies	Non-small cell lung cancer, Head and neck squamous cell cancer, Gastric adenocarcinoma, Gastroesophageal junction adenocarcinoma, Esophageal cancer	IHC	*PD-L1*
19	therascreen EGFR RGQ PCR Kit	QIAGEN	Non-small cell lung cancer	Real-Time PCR	*EGFR*
20	therascreen FGFR RGQ RT-PCR Kit	QIAGEN	Urothelial carcinoma	rRT-PCR	*EGFR*
21	therascreen KRAS RGQ PCR Kit	QIAGEN	Rectal cancer, Non-small cell lung cancer	Real-Time PCR	*KRAS*
22	therascreen PIK3CA RGQ PCR Kit	QIAGEN	Breast cancer	Real-Time PCR	*PIK3CA*
23	VENTANA anti-ALK(D5F3)	Roche Diagnostics	Non-small cell lung cancer	IHC	*ALK*
24	VENTANA anti-HER2/neu(4B5) Rabbit Monoclonal primary antibody	Roche Diagnostics	Breast cancer	IHC	*HER2*

25	VENTANA HER2 Dual ISH DNA Probe Cocktail	Roche Diagnostics	Breast cancer, Stomach cancer	ISH	*HER2*
26	VENTANA PD-L1 (SP142) Assay	Roche Diagnostics	Urothelial cancer, Non-small cell lung cancer, Triple-negative breast cancer	IHC	*PD-L1*
27	VENTANA PD-L1 (SP263) Assay	Roche Diagnostics	Non-small cell lung cancer	IHC	*PD-L1*
28	Vysis ALK Break Apart FISH Probe Kit	Dowbiomedica	Non-small cell lung cancer	FISH	*ALK*
29	ZytoLight SPEC ALK Dual Color Break Apart Probe	Mirax	Non-small cell lung cancer	FISH	*ALK*
30	ZytoLight SPEC ERBB2/CEN 17 Dual Color Probe	Mirax	Breast cancer	FISH	*ERBB2*

Table S3 Summary of safety and effectiveness of the Vysis CLL FISH Probe Kit approved by the US FDA

Division	Item	Detail
Analytical performance	**Analytical Sensitivity**	Assessed as the percentage of scoreable interphase nuclei with the expected normal signal pattern<br>Normal interphase signal pattern is considered to be 2 orange signals and 2 green signals (2R2G) by Vysis LSI TP53 SpectrumOrange and Vysis LSI ATM SpectrumGreen<br>SpectrumOrange 97.98%, SpectrumGreen 98.68% sensitivity confirmed
	**Analytical specificity**	Evaluated by the proportion of signals hybridized to the correct location<br>Hybridization positions in 20 consecutive metaphase nuclear chromosomes from each of 5 samples were assessed by a total of 200 target loci per probe<br>Confirmed 100% analysis specificity
	**Normal cutoff**	Assessed as percentage or actual number of standard nuclear classes tested and specific abnormal nuclear FISH signal patterns<br>Determination of the number of abnormal signal patterns in 200 nuclei in 25 peripheral blood samples from 25 different individuals<br>The normal cutoff for 17 deletions is set at 14 per 200 cells or 7.0%
	**Precision**	Evaluated over 3 non-consecutive days using 1 of 3 lots for a total of 7-9 repetitions<br>Ensure acceptance criteria are met for all positive and negative specimens
	**Reproducibility**	After randomization of 8 positive samples, 8 researchers evaluated in 3 laboratories<br>Repeat twice a day for 5 days based on 4 specimen types (normal, near-cutoff positive, low positive, high positive)<br>Comparison between lots and laboratories

**Robustness**		Analytical procedures (peripheral blood processing with hypotonic solution, CLL blood processing, slide preparation conditions, slide baking, temperature and time, slide maturation, slide denaturation—including ethanol dehydration and ethanol denaturation, followed by dehydration—and manual probe/slide warming/hybridization temperature) and qualitative/quantitative evaluation of time, sealing, and cleaning temperature and time<br>Confirmed 100% agreement with expected results in all studies
**Optimization and limits of probe concentration**		Evaluated by 2 researchers for 4 concentrations (2x, 1x, 0.5x, 0.33x)<br>100% confirmation at all concentrations except 0.33x
	Photostability	Evaluate white fluorescent light exposure for up to 72 hours at room temperature<br>100% confirmed in light exposure by hour (0/3/8/24/48 hours); 67% confirmed in 72 hours
	Hybridization success rate in peripheral blood	Total number of slides tested (45) and slide count evaluation to determine overall passing score for slide quality; 100% hybridization success rate confirmed
**Stability**	Sample stability	When testing immediately processed whole blood, or to evaluate the possibility of storage and delivery at 2-8°C for at least 96 hours<br>Confirm maintenance of stability after calculating the ratio of intact cells by repeating 9 times per 2 sample time points
	Cell pellet stability	Evaluate whether fixed pellets can be stored at –20°C (±5°C) for up to 24 months<br>Evaluation of 4 (normal blood, normal CLL, 2 abnormal 17p) samples at each time point (baseline, multiple midpoints, 25/26/31/35 months)<br>Ensure that results at all midpoints and endpoints are 100% consistent with baseline
	Hybrid slide safety	Stability evaluation of hybridized storage slides for more than 3 weeks at –20°C protected from light<br>A total of 18 slides were stored 3 times at –20°C for 3 weeks/6 weeks and confirmed to be 100% consistent with the baseline

		Kit stability	Stability evaluation of 12-month validity period and dry ice delivery conditions<br>Evaluated at –20°C (±5°C) for probe and DAPI II, 20°C (±5°C) and 30°C (±2°C) for 20X SSC salt and NP-40<br>Check a total of 3-9 lymphocyte slides for acceptable Q-score criteria per lot per condition
		Stability during use	Evaluation of stability maintenance after multiple freeze/thaw cycles under simulated customer use conditions<br>Confirmed 100% pass rate after evaluating 3 replicate lymphocyte slides before and after 21 freeze/thaw cycles
		Reagent Stability	Evaluation to check the expiration date of reagents in use<br>A 100% pass rate was confirmed after evaluation every hour after the expiration date with a total of 3 slides and 1 negative control per lot
	**Microorganism interference reaction**		Evaluate Q score by inoculating selected microorganisms (103-104 CFU/mL), store for 32 days and conduct performance evaluation again<br>Confirmed 67% pass rate, which is the specified standard for all test conditions (Candida albicans, Aspergillus brasiliensis, Escherichia coli, Pseudomonas aeruginosa, Pseudomonas species, Staphylococcus aureus, saline)
**Clinical performance**	**Study design**	Design	Phase 2, multicenter, single-arm study completed at 3 sites in Florida, Belgium, and Australia to evaluate the clinical performance of the Vysis CLL FISH probe kit to help identify patients in need of VENCLEXTA (venetoclax) treatment (M13-982)
		Inclusion criteria	CLL patients over 18 years of age who give informed Consent; 17p deletion confirmed by FISH kit<br>EOCG score of 2 or less<br>Normal bone marrow function, normal kidney/liver function<br>Women of childbearing age: Contraceptive from the initial drug administration to 30 days after the last administration, women negative for pregnancy<br>Men who consent to sperm donation from the initial drug

			administration to 90 days after the last drug administration<br>In the case of high-risk subjects, those who have secured prior approval
		Exclusion criteria	Handling, storage and delivery not in accordance with instructions<br>Patients who previously received VENCLEXTA (venetoclax)<br>Allogeneic stem cell transplant patients<br>Patients with prolymphocytic leukemia<br>Patients with active or uncontrolled autoimmune cytopenia<br>Human immunodeficiency virus-positive patient<br>Patients receiving anti-tumor biological agents within 8 weeks before the first drug administration<br>Patients who received anti-cancer therapy, including chemotherapy or radiation, or therapy, including targeted small molecule agents, within 14 days before the first drug administration<br>Patients receiving steroid therapy, cytochrome P450 3A inhibitors, CYP3A inducers, warfarin, or antiretrovirals within 7 days before the first drug administration
		Follow-up schedule	Follow-up visits performed approximately 30 days after discontinuation of medication due to side effects
		Clinical endpoints	Overall response rate (ORR)
	**Population**	Population characteristics	Age, race, gender, and ECOG statistical analysis
		Sample size	Of the 167 people selected as a cohort, 106 out of 144 with abnormal 17p results were enrolled
**Evaluation results**	**Safety**		The most common reactions to VENCLEXTA (venetoclax) are neutropenia, diarrhea, nausea, anemia, upper respiratory tract infection, thrombocytopenia, and fatigue

**Effectiveness**	80.2% overall response rate (ORR) confirmed in 106 patients with 17p deletion<br>Supports the basis for the purpose of use of the device through analysis performance research
**Risk–benefit analysis**	Confirmation that the test is performed consistently and provides clinical benefit for evaluating previously treated CLL patients considering use of VENCLEXTA<br>Inappropriate treatment decisions may be made if the device does not perform as expected, or test results are not interpreted accurately<br>The core of the device is the diagnostic assessment of patients with CLL in making decisions regarding treatment with VENCLEXTA, so that the expected benefits exceed the expected risks

Table S4 Summary of safety and effectiveness of the Vysis ALK Break Apart FISH Probe Kit approved in JP PMDA

Division	Item		Detail
Performance	Analytical Sensitivity		Assess the percentage of chromosomal targets showing the expected normal signal pattern<br>Signals from Vysis LSI 3'-ALK Spectral Orange Probe and Vysis LSI5'-ALK Spectral Green Probe were counted for each metaphase chromosome<br>100% confirmed analytical sensitivity of each probe
	Analytical specificity		Evaluation of the proportion of signals that hybridize only to the target locus<br>Counting the number of metaphase chromosomes showing the expected signal pattern across a total of 120 metaphase chromosomes<br>Confirmation of 100% analytical specificity of the probe
	Reproducibility	Control slide	Manual processing: Evaluation of 50 nuclear signals after counting the number of fused signals, isolated orange signals, and green signals by 3 researchers over 5 days for 3 lots each of negative and positive controls, no statistically significant differences (Fisher–Freeman–Halton test)<br>VP 2000 processing: 100% confirmed after 5 signal counts over 5 days for negative and positive controls in 3 laboratories
		Interlaboratory	Manual processing: 97.64% overall agreement confirmed after 5 days of measurements by 2 researchers in 3 laboratories using 12 specimens and 3 lots of kits<br>VP 2000 processing: 99.25% overall agreement confirmed after 3 repeated measurements by 1 researcher each over 5 days in 3 laboratories for 18 samples

	Tissue	From 20 NSCLC FFPE specimens, 3 were positive and 3 were negative, evaluated by two researchers<br>100% confirmation between researchers and slides<br>Fisher–Freeman–Halton test confirmed to be good, with P value of 1.00
	VP 2000 pre-treatment	Measurement using 3 lots of VP 2000 pre-treatment kit and VP 2000 processor, 1 lot per day for 3 days<br>Confirm 100% overall agreement with expected results, including differences between lots
	Concordance	Evaluation of agreement between manual slide processing and VP 2000 slide processing in three external laboratories<br>An average overall match rate of 95.66% was confirmed through 235 samples
**Non-specific reaction**		Lymphocyte sample: Check the size of non-specific fluorescent particles or uniform fluorescent staining in the nucleus or metaphase<br>FFPE or negative specimen: Check whether the size of non-specific fluorescent particles or uniform fluorescent staining of interphase nuclear stained cells is observed
**Probe signal strength**		Evaluation of sensitivity to distinguish between nucleus and chromosome of LSI/CEP signal in targeted hybridization

**Slide background**		Evaluate the adequacy of light/dark distinction without fluorescent particles or blur in the target area
**Cross-reaction**		Lymphocyte sample: Evaluation of cross-reaction of fluorescent signals of LSI/CEP probes in metaphase nuclei of target region<br>FFPE or negative specimen: Identification based on whether approximately equal numbers of extraneous signals are seen in the nucleus
**Microbiological test**		Subculture after adding actual microorganisms confirmed that there were no problems with the performance of the reagent, even after culturing for 3 days at 35-37°C
**Stability**	Photostability	100W mercury lamp recommended<br>A type equipped with a 10-25x objective lens and a 10x eyepiece is recommended
	FFPE fixation method	Exposure to acids such as deliming agents, strong bases, and extreme heat may cause DNA damage and is therefore not recommended<br>Set optimal fixation time (6-48 hours)
	Reagent stability	Setting the storage temperature and expiration date of Hemo-De (room temperature, 7 days)<br>Setting the storage temperature and expiration date of the pretreatment solution (37±1°C, 1 day)
	Hybrid	Denaturation (73°C, 3 minutes), hybridization (37°C, 14-24 hours) temperature and time settings

	Slide cleaning	Setting the storage temperature and expiration date of Wash Buffer I (room temperature, 1 day)<br>Setting the storage temperature and expiration date of Wash Buffer II (74±1℃, 1 day)<br>Simultaneous cleaning of 4 slides to maintain appropriate temperature of Wash Buffer II
	Dyeing	After adding DAPI I dye solution, set the light shading time (at least 5 minutes) and storage temperature (-20±10℃)
	**Concordance test using clinical specimens**	Using FFPE tissue samples from NSCLC patients enrolled in Pfizer's Phase II clinical trial protocol A8081005<br>Check positive and negative agreement rates between control group and device<br>Population characteristics: age, gender, race, ECOG stage, smoking status, disease stage, tissue classification, number of metastatic treatments obtained
**Clinical significance**	**Clinical trial**	International joint trial (A8081001): A non-blinded, single-arm, multicenter study including 15 Japanese patients treated with crizotinib alone in patients with ALK fusion gene-positive advanced non-small cell lung cancer (compare with the mainly used laboratory measurement system and check the match rate)<br>International joint trial (A8081005): Unblinded, single-arm, multicenter, phase 2 study including 6 Japanese in 133 cases treated with crizotinib alone in patients with ALK fusion gene-positive advanced non-small cell lung cancer

**Equivalence test**	Domestic phase I/II trial to evaluate the efficacy of alectinib in ALK fusion gene-positive non-small cell lung cancer patients<br>Confirmed 100% equivalence with IHC, FISH, and RT-PCR testing methods<br>Population characteristics: obtain information on organization type, gender, and age
**Brigatinib phase III international joint trial**	Evaluation of progression-free survival in 275 patients with ALK fusion gene-positive non-small cell lung cancer who had no history of treatment with ALK inhibitors<br>Crizotinib 9.8 months, risk ratio 0.49 confirmed

Table S5 Summary of safety and effectiveness of the PATHVYSION HER-2 DNA Probe Kit approved in KR MFDS

Division	Item	Detail
Analytical performance	Hybridization efficiency	Use a cell line expected to have the best hybridization efficiency, with an average percentage of cells that did not show a hybridization signal in the ProbeChek quality control slide of 0.0 to 2.0%<br>Expected efficiency is set to 98%, and if there is no hybridization reaction, set to less than 2%
	Analytical sensitivity	The average score was 1.05 (±0.03) for samples with a HER-2/neu:CEP17-ratio of 1.0 to 1.2 and an average of 1.81 (±0.08) for samples with a ratio of 1.6 to 2.0<br>The upper limit of the 95% confidence interval for samples with a HER-2/neu:CEP17-ratio of 1.0 to 1.2 is set to 1.11, and the lower limit of the 95% confidence interval for samples with a 1.6 to 2.0 ratio is set to 1.65<br>The detection limit for this product in interphase cells is set to 1.5
	Analytical specificity	Conducted using metaphase cells of normal lymphocytes according to the standard quality control method of Abbott Molecular Inc<br>The location of chromosome 17 and the HER-2/neu gene in 254 metaphase cells was continuously evaluated using the G-banding method, followed by FISH<br>Confirmed that no cross-hybridization reaction with loci of other chromosomes occurred in all 254 cells
	Reproducibility and repeatability of preclinical studies	Evaluation of serial tissue sections of various thicknesses of normal breast tissue and breast tissue with amplified HER-2/neu gene<br>The average ratio of HER-2/neu:CEP17 was 1.19 (S.D.=0.05) in a specimen obtained by serially cutting one normal breast tissue into 10 pieces, and one breast tissue with amplification of the HER-2/neu gene was cut into 10 consecutive pieces. The average ratio of HER-2/neu:CEP17 in cut specimens was confirmed to be 3.61 (S.D=0.50)<br>The average HER-2/neu:CEP17 ratio for 8 normal breast tissue samples cut sequentially at different thicknesses (2-8 ㎛) was confirmed to be 1.15 (S.D.=0.16), demonstrating the acceptable range for reproducibility

	**Control slide precision**	Compare the ratio analysis results of LSI HER-2/neu and CEP17 between laboratories, between lots, between days, and between examiners using control slides with different amplification levels<br>Full hybridization using FFPE of one breast tumor tissue with a HER-2/neu:CEP17-ratio in the normal range (1.0~1.2) and three breast tumor tissues with amplified ratio (1.6~2.0, 3~5, 7~11)<br>Success rate confirmed at 98.3%
	**Portability**	A randomized, comparative study was conducted at 5 different institutions using FISH using 4 samples (normal 1, low 2, medium 1) with different amplification levels of the HER-2/neu gene from human breast cancer tissue (FFPE)<br>There was no statistical difference in p-value in the analysis between days, and there was a significant difference in the analysis between laboratories, but it was confirmed to have no clinical relevance<br>Confirmation of 100% hybridization success rate for 5 different organs, proving convenience of use
**Clinical performance**	**CALGB 8869 clinical trial**	Tissue samples from 572 randomly selected patients were analyzed by FISH using a DNA probe to determine progression-free survival and overall survival in patients with stage 2 lymph node metastasis-positive breast cancer receiving CAF adjuvant chemotherapy<br>Determine whether the presence or absence of gene amplification can provide information on independent prognosis prediction and whether this is statistically significant<br>As a result of analyzing the disease-free survival rate of patients with HER-2/neu gene amplification confirmed by FISH testing using the Cox proportional hazards model, there was a statistical correlation between HER-2/neu gene amplification and CAF dose
	**Concordance with the test method (CTA) used in clinical research**	Comparison of test results used for patient selection in clinical studies related to Herceptin administration (H0648g, H0649g, H0650g) with test results using the PathVysion kit<br>To confirm the consistency between FISH and the test method (CTA) used in clinical research, 623 samples (317 positive, 306 negative) were randomly selected and used<br>Confirmation of 82% (95% CI, 79~85%) agreement between the two testing methods for 529 samples obtained through FISH

Table S6 Summary of safety and effectiveness of the CRCdx RAS Mutation Detection Kit approved by the US FDA

Division	Item		Detail
Analytical performance	Accuracy		Comparison of agreement with FDA-approved product (Praxis Extended RAS Panel, NGS) (n=398)<br>Evaluation of positive agreement rate (PPA) and negative agreement rate (NPA)<br>PPA, NPA evaluation by biomarker (KRAS, NRAS) determined by Praxis Extended RAS Panel NGS detection rate by mutation type of biomarkers (KRAS, NRAS)<br>Evaluation of NGS detection rate by tumor stage (I~IV)<br>Evaluation of expected tumor content rate<br>Wilcoxon signed sequence test results to assess population bias during NGS analysis<br>NGS and CRCdx agreement rate (NPA, PPA) evaluation
	Analytical Sensitivity	Limit of blank (LoB)	Analysis of FFPE samples from five wild-type CRC patients by biomarker (KRAS, NRAS)<br>Set internal control standard Ct maximum allowable range to approximately 24<br>Evaluation of 3 reagents and 8 reactions<br>Evaluation of overall negative concordance rate (wild type of KRAS, NRAS)
		Limit of detection (LoD)	First dilution: Estimated to be the lowest mutant allele frequency (MAF) with >95% identity in CRC FFPE DNA cell line, repeated 20 times with 2 lots<br>Second dilution: CRCdx evaluation at LoD greater than 6.25% MAF, 20 replicates performed in 2 lots<br>Clinical specimen confirmation: Confirmed that 16 target specimens from over 600 specimens met LoD requirements, repeated 20 times with 2 lots<br>Evaluation of dilution rate (% mutation) of 16 samples with approximately 95% or more positive

Equivalence	Linear regression analysis within acceptable prediction interval values and detection rate above 95%<br>Cell lines, FFPE cell lines, and synthetic DNA fragment samples all confirmed to be within predicted values or Ct acceptance criteria<br>Confirmation of 100% detection rate and verification of equivalence in all samples
DNA input range	Check internal control correlation coefficient and reaction efficiency range<br>Detection rate evaluation by input load (48ng, 24ng, 12ng, 6ng, 3ng, 1.5ng)<br>100% detection rate at 48/24/12/6ng, 99.3% at 3ng, and 97.1% at 1.5ng
Minimum tumor content	Use of samples with various proportions close to 20%<br>Evaluation of 391 FFPE specimens with tumor content 18-100%<br>A 100% agreement rate was confirmed in samples with a tumor content of approximately 20% (18-25%) and a 97.9% agreement rate in samples exceeding 25%
Curl number	Quantification of DNA isolated from 8 curls and dilution to 4 ng/ul<br>Dual evaluation with one reagent lot at 24 ng<br>Confirm 100% match rate for all mutations within all curl numbers
DNA extraction method characteristics	Perform DNA extraction in combination with the Maxwell CSC DNA FFPE kit<br>Performed 20 repeat experiments on 16 target samples; generate LoD samples by diluting to previously determined LoD<br>Calculate positive (99.8%), negative (95.2%), and overall (98.7%) match rates of over 95% for all clones

	Cross-reactivity (primer and probe specificity)	Evaluation of the specificity of CRCdx primers and probes to ensure that only KRAS and NRAS are amplified, and other similar genes are not amplified<br>No non-specific amplification identified by CRCdx in silico modeling software<br>No single-nucleotide polymorphisms were identified in the primer or probe binding region, which could be a false positive
**Analytical specificity**	Cross-reactivity (exclusivity)	Duplex analysis with 3 reagent lots from 35 synthetic samples<br>Evaluate at the highest acceptable Ct value (24.0) to best identify cross-reactivity<br>Confirmation of lot-specific response in repeated experiments of KRAS G12D and G12S
	Interference reaction	Evaluation of PPA, NPA of CRC FFPE specimens according to necrotic tissue content (30-70%) determined by pathologist<br>100% confirmation of PPA and NPA in all samples<br>Evaluated at concentrations exceeding 10 times those of potentially interfering substances<br>Detection confirmed in 98.4% of samples, excluding 3 samples (excess of paraffin/blue dye, excess of RNase A)
**Precision**		Performed under within-lot, within-experiment, within-user/day, between-user, between-lot, and between-site conditions<br>Dilute each mutant sample 1 x LoD, 1.5 x LoD, 3 x LoD<br>Evaluation of range and average Ct value of positive control group<br>Use precision variables with CV less than 5.0%, 0.35-3.37% within lot, 0.09-1.53% within performance, 0.11-4.54% within user/day, 0.19-2.15% between users, 0.30-3.34% between lots, 0.35-3.37% between locations

**Reproducibility**	Evaluation of inter-site and intra-site variability<br>Evaluation of reproducibility of PPA and NPA for each mutation<br>100% match rate for all mutations confirmed<br>Ct variability by site and Ct range by LoD
**Guard banding (tolerance limits)**	Evaluation of thermal cycling profile: Due to the nature of PCR, the 95℃ denaturation step and 60℃ extraction step confirm robustness to heat (temperature changes by ±1℃), and confirm 100% detection rate<br>Reaction composition evaluation: Check the robustness of the modified detection reagent + primer / probe + DNA composite (concentration ±3% change), check 100% detection rate<br>Proteinase K evaluation: Evaluation of the effect on mutation occurrence according to the amount of proteinase K (±20% correction); confirmation of 100% detection rate
**Cross-contamination**	Evaluation of cross-contamination reactions due to carryover between wells containing highly positive samples and NTC wells<br>Evaluation in the high MAF range (45.1-70.6%) by 3 investigators in 2 discontinuous checkerboard plate formats<br>Confirm 100% detection rate

	**Stability**		Stability of DNA extracted from FFPE specimens: Frozen for up to 35 days (-15~-25°C)/Refrigerated for up to 9 days (2~8°C)/Freeze-thawing evaluation for up to 3 weeks, confirming 100% detection rate<br>Open vial stability: Evaluated after initial opening and refrigerated storage (2~8°C); confirmed 100% detection rate<br>Shelf-life stability: Evaluated using reagents thawed at room temperature (25°C) for 0, 1, 2, 3, 4, 8, 12 months at specified times; confirmed 100% detection rate<br>Freeze-thaw stability: Store the reagent frozen (-15~-25°C) and evaluate it after thawing at room temperature (18-26°C) at each time point (0, 1, 2, 3, 4, and 5 weeks); 100% detection check rate<br>Master Mix stability: Evaluate after preparing Master Mix by mixing mutation reagent + primer / probe + water at the concentration that can best indicate false-positive reaction (3x, 9x LoD); confirm 100% detection rate<br>Plate stability: Mix mutagenesis reagent + primer/probe + water + DNA and distribute to 96-well reaction plate for evaluation; confirming 100% detection rate<br>Transport simulation: After processing the reagents in 4 ways (no treatment, refrigeration for 24 hours, room temperature for 24 hours, extreme heat treatment for 24 hours), thaw at room temperature to prepare Master Mix and check 100% detection rate
**Clinical performance**	**Research design**	Design and criteria	Concordance study to prove non-inferiority with the FDA-approved product 'Praxis Extended RAS Panel' to infer the efficacy of Vectibix treatment results<br>Inclusion criteria: primary colorectal cancer, adenocarcinoma of the colon or rectum, 90% white, average age 60-65 years, 500 subjects, various tumor subtypes and stages, male or female over 18 years of age, signed informed consent, 10% neutral formalin and FFPE samples prepared for 24 hours, median area of sample tissue at least 40 but less than 300, median tumor content 45-55%

		Exclusion criteria: samples that did not meet the inclusion criteria, samples that failed DNA pre-qualification
	Follow-up	Not applicable, as it included a retrospective study
	Clinical validity	Evaluated 398 CRC FFPE specimens using non-inferiority statistical analysis
**Population**	Population characteristics	Comparison of patient population characteristics between Praxis and CRCdx<br>Gender, age, ethnicity, mutation status, tumor stage, tumor content, tissue sample
	Sample size	Total number of samples by tumor stage<br>Tumor content distribution<br>Wilcoxon signed sequence test results to identify population bias<br>Prevalence of genotype
**Evaluation results**	**Effectiveness**	Demonstrated in a retrospective concordance study using CRC patient FFPE tissue with a previously approved device<br>Concordance between the two devices supports the kit's effectiveness in identifying patients with CRCdx results whose tumors are positive for variants listed in the intended use and for use of relevant therapeutic agents
	**Safety**	There is a possibility of errors in patient management due to incorrect test results<br>False-positive or false-negative results may result in treatment being withheld or related side effects occurring<br>Proven safety for use of the device due to analytical performance tests

**Risk–benefit decision**	Although it showed a clinically significant benefit through a study consistent with existing approved products, the NPA was 92%, so there is a possibility of clinical false negatives, but the false-positive rate is low at 0.4%, so it is judged to be an acceptable false negative<br>Demonstrate non-inferiority and demonstrate clinical benefit of the kit<br>There is a risk of false positives, false negatives, failure to provide results, and misinterpretation by the user, which may result in treatment being withheld or potential side effects occurring<br>However, the risk is mitigated by the analytical performance of the device and the performance of clinical non-inferiority studies<br>In conclusion, considering the overall data, the expected benefits of using CRCdx to help identify patients eligible for Vectibix treatment exceed the expected risks

Table S7 Summary of safety and effectiveness of the therascreen EGFR RGQ PCR Kit approved in JP PMDA

Division	Item	Detail
Performance	**Control Ct value**	Confirmation of Ct value using positive and negative control samples<br>Positive: Control 28.38-34.34, T790M 30.47-34.73, Deletions 29.15-34.65, L858R 30.22-34.56, L861Q 28.74-33.74, G719X 29.67-33.94, S768I 29.23-34.94, Check insertions 28.17-33.84<br>Negative: 8 total responses 29.85-35.84 confirmed
	**Minimum detection limit**	Calculation of minimum concentration detectable as positive at 95% using FFPE clinical specimens and FFPE+plasmid DNA specimens<br>Each LoD is calculated by logistic regression analysis
	**Cross-reaction**	As a result of analysis with high-concentration DNA FFPE samples, the minimum Ct value was higher than each Ct value in all samples, so cross-reactivity was not recognized
	**Accuracy**	By comparing this product with the reference method, the Sanger method, an overall agreement rate of 92.2% was confirmed
	**Precision and reproducibility**	Two researchers evaluated two instruments in three laboratories using DNA extracted from NSCLC FFPE tissue, FFPE cell line, and wild-type FFPE tissue<br>As a result of double measurement of 3 lots for 16 days, total coefficient of variation was confirmed to be less than 14.11%, coefficient of variation between lots/days/measurements was less than 8.33%, and simultaneous reproducibility was confirmed to be 5.99-13.49%
	**Correlation**	Using 192 samples, an overall consistency rate of 97.4% was confirmed between this product and existing approved products
	**Interference reaction**	Evaluation of the impact of harmful substances (paraffin, xylene, ethanol 96-100%, proteinase K) that may affect measurement<br>Confirmed that there is no effect up to 10 times the concentration of each substance

**Clinical significance**	**Afatinib maleate clinical study**	Retrospective study based on the results of a randomized, multicenter, phase 3 trial (CTA) in patients with EGFR mutation-positive lung adenocarcinoma treated first-line with afatinib and chemotherapy<br>Progression-free survival (PFS) was confirmed to be significantly prolonged in the afatinib treatment group compared to the chemotherapy group (11.2 months vs 6.9 months, hazard ratio 0.49)
	**Gefitinib clinical study**	Gefitinib as first-line treatment in Caucasian patients (stage IIIA/B/IV) with EGFR mutation-positive locally advanced or metastatic NSCLC by single-arm, unblinded IFUM trial (Iressa follow-up measures study: phase VI trial)<br>Patients were retrospectively screened for deletions in EGFR exon 19, L858R, L861Q, substitutions in G719X, no mutations in T790M and S768I, and insertions in exon 20<br>Confirmed overall (98.2%), positive (88.2%), and negative (99.8%) agreement rates with CTA in the detection of exon 19 deletion and L858R mutation<br>ORR was assessed by random independent central review (BICR) and clinicians, and confirmed by BICR in 48.3% and by clinicians in 71.3%<br>Due to additional validity interpretation with CTA, it is equivalent and evaluated as the interpretation results of clinical trials
	**Dacomitinib clinical study**	An international collaborative phase III trial targeting non-small cell lung cancer patients with EGFR gene mutations without a history of chemotherapy<br>PFS evaluation after randomization of 227 samples in the dacomitinib group (40 Japanese people) and 225 in the gefitinib group (41 Japanese people) among a total of 452 samples (81 Japanese people)<br>14.7 months in the dacomitinib group and 9.2 months in the gefitinib Group; there was a statistically significant increase compared to gefitinib (HR=0.589, P<0.0001)<br>By evaluating the concordance rate between CTA of EGFR mutation-positive patients and this product, the overall concordance rate was confirmed to be 96.5%

Table S8 Summary of safety and efficacy of the therascreen KRAS RGQ PCR Kit approved in KR MFDS

Division	Item	Detail
Analytical performance	**Cut-off**	Analysis of 220 samples using methods guided by NCCLS EP17-A<br>Confirmed control Ct range 21.92-32.00<br>Set based on the Ct of the mutation reaction minus the Ct of the control reaction
	**Limit of blank (LoB)**	Evaluate template-free samples to ensure they do not produce analytical signals that may indicate low-abundance mutations<br>None of the mutant or control reaction tubes showed detectable control or mutant Ct values
	**Comparison with standard methods**	Evaluation of 350 tumor specimens obtained based on baseline clinical, demographic, and tumor specimen characteristics<br>Confirmed 96.8% agreement with bidirectional sequencing analysis
	**Limit of detection (LoD)**	Testing 9 replicates per dilution, creating a mid-range control Ct value (approximately 26)<br>Determine the proportion of correct decisions as a function of dilution in low dilution series
	**Influence of input DNA**	The performance of the kit is proven to be consistent across the total DNA input (control Ct) range of the assay<br>There is an increase in Ct values as DNA input increases, but consistency is confirmed to be within the specified acceptance criteria
	**Linearity/ amplification efficiency**	Prove the linearity and amplification efficiency of PCR in each mutation reaction by comparing it with the control reaction<br>Compared to the mutation reaction, the amplification efficiency of the control group confirmed that ΔCt was consistent across the range of analysis items<br>Evaluate the impact of serially diluted mutation-positive samples on amplification efficiency at an input level of approximately 22-23 Ct

**Interfering substances**	Evaluate the impact of potential interfering substances (paraffin wax, xylene, ethanol, buffer ATL, proteinase K, buffer AL, washing buffer) on the performance of the kit<br>Verification that none of the potentially interfering substances evaluated at concentrations expected to occur during normal use will affect performance
**Cross-contamination**	Use kits to measure the degree of cross-contamination between DNA samples, potentially leading to false-positive results<br>Confirm that no contamination is detected in sample extraction, pipetting, tube closing, kit reagent contamination, or analysis item tube loading
**Cross-reaction**	Evaluated at the level of highest concentration to address the amount of non-specific amplification of wild-type DNA by reaction mixtures designed to amplify specific mutations<br>Check for undetected or out-of-threshold results in all cases
**Repeatability, reproducibility**	Two practitioners evaluate all samples and controls with 3 lots of kits twice a day for 5 days<br>Ensure accurate mutation determination of at least 39/40 across all parameters within and between laboratory experiments and across multiple lots, platforms, and operators
**Sample processing variability**	Comparison of mutation determination and average Ct values of wild-type samples by site<br>Confirmation of 99.33% agreement between 3 sites related to mutation determination
**Lot Interchangeability**	Using three lots, three independent lots were each evaluated with six replicates, with samples having target control values of approximately 23, 26, and 31

**Clinical performance**	**Cetuximab**	The safety and effectiveness of cetuximab were demonstrated in the CA225025 study (a multicenter, open-label, randomized clinical trial conducted in 572 patients with previously treated EGFR-expressing recurrent metastatic CRC)<br>Samples from 453 of 572 patients are used in clinical trials<br>The overall survival time, which is the main outcome measure, was confirmed to be higher in the treatment group combined with cetuximab than the control group

Table S9 Summary of safety and effectiveness of the Praxis Extended RAS Panel approved by the US FDA

Division	Item		Detail
Analytical performance	Accuracy		Comparison of agreement with the reference method, Sanger sequencing, using FFPE CRC tissue samples collected and stored during the Amgen Panitumumab study (protocol 20050203) (n=1,183)<br>Evaluation of positive and negative agreement rates (PPA, NPA) for sample-level comparison<br>Evaluation of positive and negative agreement rates (PPA, NPA) to compare mutation (KRAS, NRAS) levels
	Analytical sensitivity	Limit of blank (LoB)	A total of 60 mutations observed per lot for 3 wild-type samples (20 x 3 samples)<br>Set clinical threshold (2.6%) after analyzing samples without somatic mutations ('Blank')
		Limit of detection (LoD)	Determination of the lowest allele frequency among samples detecting more than 95%<br>Evaluation at 4 (10/5/2.5/1.25%) mutation frequency levels by 5 researchers using 2 reagent lots over 2 non-consecutive days<br>Establishing LoD ranges for six representative mutations using probit analysis (4.2-5.3%)
		DNA input	Evaluation of changes in quantification threshold (Cq) between sample and control template DNA considering the quantity and quality of samples for DNA qualification<br>DNA input evaluation according to Cq range (-0.5~5) to evaluate acceptable measurement value (Delta Cq ≤ 5)<br>Evaluation divided into upper limit (-0.5~0.5) and lower limit (4~5)<br>Additional analysis in the range of dCq 6-4 to demonstrate performance on challenging samples near LoD<br>Check the accuracy of DNA input corresponding to dCq ≤ 5 and the amount of intact DNA of the mutation near LoD (approximately 25 ng)

**Analytical specificity**	Interference reaction (exogenous substances)	Evaluation of 9 exogenous substances (paraffin removal solution, paraffin wax, xylene, ethanol, proteinase K, cleaning solutions from 3 commercial extraction kits.) to assess the potential impact of interfering substances<br>False-positive and false-negative results not confirmed
	Interference reactions (endogenous substances)	Evaluation of analytical performance when samples contain a large amount of necrotic tissue (10-80% in 15 CRC FFPE samples)<br>Confirm 100% and 99.9% agreement rates by comparing Sanger sequencing with PPA and NPA<br>Confirmed 100% agreement with PPA and NPA by evaluating hemoglobin at 2 mg/ml according to CLSI EP7
**Reproducibility**		Inter-site reproducibility: 2 replicates performed at 3 sites by 2 researchers over 3 non-consecutive days<br>99.6% detection rate confirmed by frequency level (VAF) of individual unique mutations<br>Analysis of variance component estimates for individual unique mutations
**Precision**		Evaluation with 3 reagent lots in 5 mixing panels 12 observations performed with 2 instruments repeated 2 times over 3 days<br>99.7% accuracy for all lots and dates
**Equivalence (clinical samples)**		Comparison of variant detection performance in mutant allele frequency (AF) to support the use of cell lines in case it is difficult to obtain clinical specimens (clinical FFPE specimens vs. cell line FFPE specimens)<br>Set mutation frequency level (10/5/2.5/1.25%) and target delta Cq (4.5±0.5)<br>5 researchers using 2 reagent lots over 2 days, producing a total of 40 results<br>By comparing regression coefficients between clinical samples and cell lines, the difference in prediction estimates is small, providing clinically acceptable results (similar performance)

**Equivalence (FFPE extraction method)**	Performance evaluation of three commercially available kits against extraction kits not included in Praxis<br>Used 10 tissue samples and 3 commercial kits, including 8 common mutants and 2 wild-types<br>100% confirmation of OPA, PPA, and NPA for each item<br>DNA production of similar quality and quantity was confirmed as a result of deriving dCq and allele frequency from three extraction methods
**PCR thermocycler systems comparison**	Performance evaluation of three commercially available PCR thermocycler systems used in the PCR amplification step<br>Using 10 genomic DNAs from duplicate FFPE tissues, including 7 common mutants and 3 wild-types<br>100% confirmation of OPA, PPA, and NPA through Sanger sequencing of 10 samples
**qPCR system comparison**	The Praxis kit includes reagents for sample qualification (quality control primers, quality control templates, qPCR master mix), but does not specify a qPCR system, so a study to prove consistency was performed<br>Evaluation of 20 gDNA samples from FFPE tissue using pre-characterized dCq using 4 commercially available qPCR instruments<br>Sample evaluation of delta Cq value by qPCR system
**Sample carryover**	Assess whether false-positive results due to contamination between wells (within a run) and contamination between successive sequencing runs (between runs) occur in less than 1% of events<br>Carryover between wells: Run a checkerboard layout alternating between NRAS or KRAS mutations at low gDNA and KRAS or NRAS mutations at high gDNA<br>Carryover between runs: Generate high-frequency KRAS and NRAS samples and then sequence NRAS, followed by KRAS sequencing (and vice versa)

	Confirmed that all expected variants were detected and no false negatives were observed
**Specimen handling**	Temperature storage and freeze-thawing of DNA extracted from tissues, and evaluation of the impact of storage conditions on performance<br>Handling conditions: Stored at 4°C for 21 days/28 days and further time points, stored at -20°C for 62 days/76 days, freeze-thawed 2x or 3x<br>100% confirmation of OPA, PPA, and NPA for each condition and time point
**Guard banding**	Assess sample eligibility, library preparation and sequencing steps<br>Sample qualification: Five gDNA samples were included in the qPCR master mix and reaction mix and evaluated at various storage times (1 hour at room temperature, 10 minutes on ice) to confirm an average dCq difference of less than 0.25 (proven stability)<br>Library preparation: Assess robustness of hybridization, incubation time, solution storage time, heat denaturation and cooling time to ensure accuracy range of 90-100%<br>Sequencing: A total of 40 samples and controls were evaluated by two researchers to meet a minimum coverage of 1800x across the maximum multiplexing level of the assay at 10 samples per run, resulting in a confirmed coverage of 31000x
**Stability**	Open tube: Freeze/thaw stability evaluation of 6 kits to detect errors after opening reagents<br>Real-time: Using the study design described in CLSI EP25-A to establish the shelf life, three kit lots were used according to storage conditions (-15 to -25°C frozen, 2 to 8°C refrigerated, 15 to 30°C room temperature).<br>Repeat 3 times per period (0/3/6/9/12/13/15/16/18 months)<br>Transport stability: Perform atmospheric thermal cycling of frozen and refrigerated products in insulated containers to evaluate the impact of external temperature on product function

**Clinical performance**	**Research design**	Design	Retrospective study using FFPE CRC tissue samples collected and stored during the Amgen panitumumab study<br>Amgen Study: Multicenter, prospective, open-label, randomized phase 3 study in 1,183 patients with previously untreated mCRC
		Inclusion and exclusion criteria	Inclusion criteria: samples collected as part of an Amgen study, samples meeting tissue requirements, samples with a reasonable minimum tissue area (80 mm with tumor content ≥50%), and maximizing dissection performed if tumor content is <50%<br>Exclusion criteria: Samples that did not meet the inclusion criteria
		Clinical endpoints	Primary endpoint: progression-free survival rate, overall survival rate<br>Clinical feasibility: Evaluating whether PFS and OS are improved in combination treatment with panitumumab and FORFOX compared to FOLFOX treatment alone<br>Log-rank test, hazard ratio, Kaplan–Meier (KM) curve, KM estimate, number of subjects censored, and number of subjects with an event
	**Population**	Population characteristics	Subject demographics (ethnicity, gender, age) and disease characteristics (tumor type, severity, stage)
		Sampling criteria	Description of sample classifications potentially suitable for the study (FFPE samples available, panel positive patients, samples below delta threshold 5.0, valid RAS results)<br>Panel-based genetic mutation subtype classification
		Sample size	Number of subjects evaluable and non-evaluable and reasons for this<br>Number of samples by evaluable RAS gene/exon status and treatment group

<table>
<tr><td rowspan="3">Evaluation results</td><td>Effectiveness</td><td>For wild-type RAS, the hazard ratio for PFS was confirmed to be 0.700 (0.516-0.948), and statistical significance of PFS was confirmed between the panitumumab + FOLFOX and FOLFOX alone groups (p=0.0206)
For wild-type RAS, the hazard ratio for OS was found to be 0.754
For mutant RAS, the hazard ratio for PFS was 1.242, and the hazard ratio for OS was 1.156
Kaplan–Meier analysis of survival by treatment for wild-type vs. mutant
Sensitivity analysis: evaluation of multivariate models after adjusting for covariates to assess impact on results for missing data
Subgroup analysis: perform primary analysis including all samples, regardless of dCq value</td></tr>
<tr><td>Safety</td><td>No changes were observed in the safety profile for panitumumab in clinical validation studies
However, adverse reaction rates were observed in patients treated with the combination of panitumumab and FOLFOX</td></tr>
<tr><td>Risk–benefit decision</td><td>Effectiveness: In wild-type RAS subjects, the effect of panitumumab + FOLFOX is more advantageous in improving PFS than monotherapy, and the accuracy according to the analysis performance study appears to be at an acceptable level
Safety: False-positive and false-negative results may lead to treatment withholding and potential side effects, but analysis performance evaluation shows that it is safe to use in mCRC subjects
In conclusion, Praxis helps identify patients suitable for panitumumab treatment, where the expected benefits exceed the expected risks</td></tr>
</table>

Table S10 Summary of safety and effectiveness of the OncoGuide NCC Oncopanel System approved in JP PMDA

Division	Item	Detail
Analytical performance	Accuracy	Proven consistency with approved devices (Histofine ALK iAEP kit, Ventana OptiView ALK D5F3, OncoGuide AmyDx ROS1 Gene Fusions Detection Kit, Dako Hercep Test)<br>Positive/negative concordance rates were confirmed for a total of 34 samples of human epidermal growth factor receptor 2 (HER2), anaplastic lymphoma kinase (ALK), and ROS1 fusion genes<br>Check the match rate with Sanger sequencing and MassARRAY systems to evaluate the accuracy of gene substitution and insertion–deletion<br>Check the concordance rate with quantitative PCR to evaluate accuracy for detection of gene copy number changes<br>Check the concordance rate with full exon sequencing to evaluate the accuracy of TMB detection
	Precision	Evaluation based on the allele frequency of positive samples in the form of gene substitutions, insertions, deletions, copy number changes, and fusions and negative samples in the laboratory<br>Coefficient of variation estimated at 8.8-20.7% for expected allele frequency of 5%<br>Repeatability determined at 8.5-19.4% by performing 4 repetitions
	Limit of detection (LoD)	Evaluation of mutation detection rate between the lower limit (10 ng) and upper limit (200 ng) of DNA content after repeated analysis<br>Establish detection limits based on mutation detection rate using laboratory specimens that are non-tumor tissue specimens or specimens with multiple allele frequencies or copy numbers
	Organization type	Foreign substances that inhibit enzyme reactions were removed during the FFPE manufacturing and DNA extraction process, and mutations were detected in over 40 cancer samples, including rare cancers, so separate tissue types were not evaluated

	**Specificity**	Read count assessment using three commercially available samples and laboratory clinical samples enriched for target gene regions<br>99.13-99.60% confirmation for 100 or more commercial specimens and 97.02-98.68% confirmation for clinical specimens
	**Interference reaction**	There were no special problems in existing clinical studies, and the DNA extraction kit recommended for use in the device was designed to properly remove impurities so that they are not carried over to subsequent processing steps, so they were not evaluated for separate interfering substances
	**Comparison with prototype**	A 100% match rate was confirmed by comparing the positive match rate with the MassARRAY system for 53 gene substitutions, 16 insertions, 22 copy number changes, and 2 fusion samples
**Clinical performance**	**Appropriateness of the proposed target gene**	It is clinically useful to present TMB results obtained from gene panel tests used in CGP, which is designed to detect more than 100 genes because accurate measurement of TMB requires gene sequence analysis information within a region of approximately 500 kB<br>Proposed target genes for reference are classified according to the level of evidence in the academic society guidelines published in 2017<br>Target genes include more than 50 genes reported as mutations with a level of evidence 3A or higher<br>Conducted clinical research using the NCC Oncopanel prototype targeting patients with advanced or recurrent solid tumors from 2016 to 2018<br>Proof of feasibility by considering similar patient ratio and drug treatment results as the MSK-IMPACT study approved in the United States

	**Adequacy of sensitivity for detection of target mutations**	Prove adequacy with data on analysis performance (accuracy, precision, and detection limit) of mutation detection<br>Sanger sequencing and MassARRAY analysis and detection consistency rate proven to evaluate the accuracy of detection of base substitutions and insertions in mutations<br>Proof of reasons for 4 discrepant samples (samples with less than 50% tumor content) due to mutation copy number change or gene fusion<br>Demonstrate rationale for the accuracy of the prototype using NCC and other analytical software programs, library building reagents, or panel reagents
	**Preparation of result report and appropriateness of content**	Regular updating and management of used databases (clinical variants, genetic definitions, SNPs, known mutations, false-positive mutations)<br>Specifies information collection procedures, including items to be used for each DB and guidelines to be followed
	**Clinical performance**	Proven by the suitability of the proposed analysis target genes, sensitivity for detection of target mutations, and generation and content of result reports
**Evaluation results**	**Purpose of use**	Provides information on genetic mutations to help develop treatment plans based on comprehensive profiling of 114 cancer-related genes isolated from solid tumor patient tissue
	**Approval conditions**	Providing products to medical institutions so that doctors with sufficient knowledge and experience in cancer genomic medicine can determine and utilize patients' eligibility and timing for genetic testing according to the latest guidelines developed by relevant academic societies

Table S11 Summary of safety and effectiveness of the Oncomine Dx Target Test approved in KR MFDS

Division	Item	Detail
**Analytical performance**	**Limit of blank (LoB)**	Analytical performance limit of blank (LoB)—for each mutation position that can be detected using a wild-type sample in the Oncomine Dx Target Test, to demonstrate that a sample without the mutation does not generate an analytical signal that can be classified as a mutation. After evaluation, the test result is 0 in 95% of samples without variants as defined by CLSI EP17-A2<br>By examining the frequency of false positives for EGFR Exon 20 insertion detected by the Oncomine Dx Target Test in wild-type samples, the test result was confirmed to be 0 in 95% of samples without mutations according to the definition of EP17-A2
	**Tissue content**	Analyze 60 enclosed FFPE sample slides to determine whether the DNA and RNA yields extracted using the Ion Torrent Dx Total Nucleic Acid Isolation Kit produce the concentration required for the Oncomine Dx Target Test when the tissue input meets the requirements<br>Of the 60 samples tested, 98.3% (59/60) had a DNA concentration of ≥ 0.83 ng/μℓ and an RNA concentration of ≥1.43 ng/μℓ, and one CNB sample had a DNA concentration of 0.52 ng/μℓ and an RNA concentration of ≥1.43 ng/μℓ<br>Confirmed that it does not meet the minimum DNA and RNA concentration standards at 1.23 ng/μℓ<br>Seven slide-mounted FFPE fine-needle aspiration test samples were analyzed to determine whether samples extracted using the Ion Torrent Dx Total Nucleic Acid Isolation Kit at the concentration required by the Oncomine Dx Target Test contained DNA and RNA when tissue content requirements were met<br>Determine whether the extracted sample yielded DNA and RNA<br>100% of the 7 FNA samples extracted using the Ion Torrent Dx Total Sample Preparation Kit were confirmed to produce DNA with a concentration of ≥0.83 ng/μL and RNA with a concentration of ≥1.43 ng/μL

**Guard banding**	Using a mixed sample of EGFR exon 20 insertion mutations, major analysis steps were performed at each key point in the NSCLC workflow from library preparation to sequencing, and tolerance was evaluated under three test concentration conditions (low, normal, and high)<br>Confirm the acceptable range by testing 10 test conditions, such as dose, temperature, and time, using a mixed sample of DNA nucleic acid extract and EGFR insertion obtained from a clinical FFPE sample
**Stability**	Evaluate the impact on performance according to the storage time of stopping points specified in the user manual<br>There are three storage time conditions (no storage, library storage, and combo storage) to compare baseline analysis performance with DNA containing one mutation and analysis performance tested after storage at the stopping point<br>The test results showed no effect due to storage time, and 100% detection was confirmed in all clinical samples
**DNA/RNA input**	A total of 540 individual DNA and RNA libraries were tested 6 times repeatedly for each test condition, including positive control and No Template Control (NTC)<br>DNA and RNA mixture set at 15% target allele frequency for target fusion of 300-600 reads for SNVs, deletions, and ROS1 mutations<br>The test results showed a 100% positive variant call rate (variant information extraction rate) within the DNA and RNA dosage range, and the Oncomine Dx Target Test proved that it was necessary to administer 10 ng of DNA and RNA each
**Cross-reaction**	False positives for the human genome, human transcriptome, and representative bacterial, fungal, and viral genomes frequently found in human tissue and lung samples to confirm the specificity of the 827 primers included in the DNA and RNA panel for the target base sequence reaction evaluation<br>There was an unintended amplification product, but retest confirmed that it did not cause a false-positive result

**Cross-contamination**	Evaluation of false-positive rate due to cross-contamination and carryover contamination using a total of 8 FFPE cell line samples<br>Among 100 DNA and 80 RNA data points, there were no false-positive results reported for DNA mutations, and one false-positive result was reported for ROS1 fusion mutation, resulting in a DNA false-positive rate of 0% and RNA false-positive rate of 1.25%
**Tissue fixation**	Evaluation of the effect of 10% neutral buffered formalin (NBF) fixation time on cytosine deamination reaction at the targeted hotspot location<br>The pellet obtained from the wild-type cell line GM24385 was fixed with 10% NBF for 12/24/48/72/84 hours and compared with the test results of the cell line GM24385 that was not fixed with 10% NBF<br>As a result of testing all NBF fixation times, valid sequencing results for both DNA and RNA were confirmed
**Precision**	To evaluate the reproducibility and repeatability of genetic mutation detection, 4 researchers evaluated 10 samples, including 2 wild-type samples and 10 mutation-positive samples, from 4 testing institutions<br>Each sample was tested 8 times at each testing institution, and each sample was tested a total of 32 times, resulting in a final number of invalid reactions of 15/768 (1.95%)<br>The repeatability estimate for 218 mutation positions out of 605 was 100%, the repeatability estimate for 175 mutation positions out of 605 was 94-99.9%, and the repeatability estimate for 212 mutation positions out of 605 was 71.6-93.9%

**Interference reaction**	Evaluation of six potential interfering substances (paraffin, xylene, ethanol, hemoglobin, protease, wash buffer) that can be found in FFPE tissue samples or carried over during deparaffinization and nucleic acid extraction<br>Perform repeated interference testing using updated RNA library preparation workflow<br>To prove that RET fusion detection performance is not affected by potential interfering substances, a total of 3 FFPE (2 RET fusion positive, 1 wild-type) were repeated with 3 replicates<br>To demonstrate that EGFR exon 20 insertion-positive detection performance is not affected by potential interfering substances, a total of three FFPE samples (two EGFR exon 20 insertion-positive samples and one WT sample) with two replicates each were used<br>The positive and negative agreement rates compared to the control condition for each potential interfering substance used in sample extraction, excluding No call, were 100% for all samples, and the overall agreement rate compared to the control condition was confirmed to be 100%
**Minimum limit of detection**	The LoD for DNA mutations was measured to have an allele frequency of 6-8% through 10 repeated experiments per 6 or more concentration ranges in 2 lot reagents for each of the 14 mutations detected in clinical samples<br>Using an RNA workflow, two clinical ROS1 fusion-positive specimens were tested with two reagent lots, with 10 replicates per six dilution levels, resulting in 516 fusion reads<br>Using an RNA workflow, 2 clinical RET fusion-positive specimens were tested in 2 reagent lots with 10 replicates per 6 dilution levels, resulting in 405 fusion reads<br>Insertion of 2 clinical EGFR exon 20 into 2 reagent lots with 10 replicates per 6 dilution levels using workflow<br>Test positive samples to confirm allele frequency of 4.8-5.2%
**Tumor cell content**	Calculated for previously identified FFPE clinical samples of 71 ROS1 specimens, 110 EGFR exon 20 insertion specimens, and 216 RET fusion specimens<br>The corresponding 95% Clopper Pearson Exact CIs for PPA, NPA, and OPA overlapped between tumor cell content levels, confirming that they were similar across all ranges

	**Repeatability**	Evaluate intra-assay precision performance and variability across institutions, investigators, and instrument platforms using 30 representative variants from 18 DNA samples<br>Repeatability estimate for each DNA mutation position was ≥98.8% in all samples except No call, and the coefficient of variation for DNA mutation in all clinical samples was 9.8%-39%
	**Accuracy**	Evaluate agreement by comparing with validated standard testing methods (detection of SNV and deletion hotspot mutations with validated NGS assay, detection of ROS1 fusion with ROS1 FISH standard test method)<br>Confirmed OPA 100% for each mutation location excluding No call, OPA 99.4% according to mutation type, and OPA 97.6% according to each FFPE specimen
**Clinical performance**	**BRAF**	Evaluated the accuracy of the Oncomine Dx Target Test and compared it with the NSCLC BRAF V600E PCR assay to detect BRAF V600E mutation using patient samples and collected negative samples from the BRAF113928 NSCLC clinical trial<br>Valid results were confirmed in both the BRAF V600E PCR assay and the Oncomine Dx Target Test for 181 samples out of a total of 230 samples<br>Except for No call, OPA, PPA, and NPA are all 100% confirmed
	**EGFR L858R, Exon 19 deletion**	Performance evaluation of the Oncomine Dx Target Test that detects EGFR biomarkers in 92 samples obtained from patients confirmed positive using the Qiagen therascreen EGFR RGQ PCR Kit<br>Evaluation of agreement rate between Qiagen EGFR PCR assay, a standard test method, and Oncomine Dx Target Test, a standard test method, for a total of 193 samples<br>Confirmed overall match rate of 99% for EGFR L858R and 100% overall match rate for Exon 19 deletion

**EGFR exon 20 insertion**	Using clinical trial analysis (CTA), 100% OPA was confirmed after evaluating the agreement rate between the Oncomine Dx Target Test and the validated reference NGS assay for 55 samples from patients confirmed positive<br>Clinical effectiveness was demonstrated by measuring the overall response rate (ORR) of NSCLC patients with positive EGFR Exon 20 insertion mutations and confirming an objective response rate (ORR) of 47.4%
**RET**	OPA of 94.9% was confirmed after evaluating the agreement rate between NGS test methods for 203 samples confirmed as positive LLT test results<br>ORR and ORR of patients confirmed as positive for RET fusion as a result of the Oncomine Dx Target Test (ORR was calculated for patients receiving selpercatinib, a RET inhibitor after platinum therapy, and for patients receiving selpercatinib without platinum therapy, DCR, and clinical advantage ratio CBR)
**ROS1**	Evaluated the concordance rate between the Oncomine Dx Target Test and the ROS1 FISH assay by using samples from 19 patients who participated in Pfizer's phase 1 study (A8081001), which were confirmed positive through the ROS1 FISH assay, and 13 stored positive sample OPA 96.5% confirmed
